# Synucleins Antagonize Endoplasmic Reticulum Function to Modulate Dopamine Transporter Trafficking

**DOI:** 10.1371/journal.pone.0070872

**Published:** 2013-08-13

**Authors:** Adam W. Oaks, Nicholas Marsh-Armstrong, Jessica M. Jones, Joel J. Credle, Anita Sidhu

**Affiliations:** 1 Laboratory of Molecular Neurochemistry, Department of Biochemistry and Molecular & Cellular Biology, Georgetown University Medical Center, Washington, D.C., United States of America; 2 The Solomon H. Snyder Department of Neuroscience, The Johns Hopkins University School of Medicine, Baltimore, Maryland, United States of America; 3 Hugo W. Moser Research Institute at Kennedy Krieger, Baltimore, Maryland, United States of America; Ludwig Maximilians University Munich, Germany

## Abstract

Synaptic re-uptake of dopamine is dependent on the dopamine transporter (DAT), which is regulated by its distribution to the cell surface. DAT trafficking is modulated by the Parkinson's disease-linked protein alpha-synuclein, but the contribution of synuclein family members beta-synuclein and gamma-synuclein to DAT trafficking is not known. Here we use SH-SY5Y cells as a model of DAT trafficking to demonstrate that all three synucleins negatively regulate cell surface distribution of DAT. Under these conditions the synucleins limit export of DAT from the endoplasmic reticulum (ER) by impairment of the ER-Golgi transition, leading to accumulation of DAT in this compartment. This mechanism for regulating DAT export indirectly through effects on ER and Golgi function represents a previously unappreciated role for the extended synuclein family that is likely applicable to trafficking of the many proteins that rely on the secretory pathway.

## Introduction

The synuclein (Syn) family of proteins includes three paralogous isoforms alpha (α-Syn), beta (β-Syn), and gamma (γ-Syn) and represents a unique class of abundant brain proteins. α-Syn is closely associated with the neurodegenerative pathology of Parkinson's disease (PD), and is linked to both autosomal dominant [1], [2], [3], [4] and idiopathic [5] forms of the disease. Lewy bodies, a pathological hallmark of PD and other synucleinopathies, contain aggregated α-Syn [6]. β-Syn and γ-Syn have also been linked to the neurodegenerative lesions of PD [7], and γ-Syn is further connected to glaucoma as well as cancer progression [8], [9], [10]. Nonetheless, the normal function of the Syn proteins remains poorly described.

Physiologically, the Syn proteins have been defined as modulators of synaptic function, with many proposed mechanisms of action, including chaperoning of membrane fusion complexes and maintenance of the synaptic integrity that is essential for neurotransmission [11], [12]. Though Syns exist largely as freely diffusing unstructured proteins, they do possess membrane-binding characteristics that could allow interactions with pre-synaptic vesicles, linking the Syn proteins to regulation of the storage, exocytosis, and release of neurotransmitters [13], [14], [15], [16], [17], [18], [19], [20]. We have shown that an important function of the Syns is the regulation of the synaptic content of neurotransmitters through modulation of monoamine transporter (MAT) re-uptake activity, trafficking, and plasma membrane distribution [21]. As MAT are the sole determinants of neurotransmitter re-uptake, the modulation of the transporters of dopamine (DAT), norepinephrine (NET), and serotonin (SERT) by the Syn is an important neuroregulatory function that has implications for PD as well as neuropsychiatric disorders, including depression, anxiety, sleep and attention deficit disorders, and drug addiction.

We have previously shown that trafficking and function of NET is modulated by α-Syn, β-Syn, and γ-Syn [22], [23], while SERT is modulated by α-Syn and to a lesser extent by γ-Syn [24], [25]. The modulation of DAT by α-Syn is well described *in vitro* and is mediated through direct protein-protein interactions between the NAC (non-amyloid-β component) region of α-Syn and the final 22 amino acids of the DAT carboxy terminal [26], [27], [28], [29], [30], [31]. Nonetheless, efforts to confirm this functional relationship *in vivo* have produced mixed results, as several studies have shown that deletion of α-Syn has little [32] or no [13], [16], [33] effect on DAT distribution and activity in the mouse brain. Other work has shown that lentiviral-mediated over-expression of α-Syn potentiates the behavioral response to cocaine, which is a DAT-mediated function [34]. Though intriguing, these *in vivo* studies provide no direct evidence for α-Syn trafficking of DAT and raise the possibility that compensatory mechanisms, possibly involving the remaining Syn proteins, mask the predicted *in vivo* function of α-Syn.

To date, the possible contribution of β-Syn and γ-Syn to DAT function and trafficking has not been analyzed, nor has the precise mechanism by which α-Syn modulates DAT been defined. It is therefore of great interest to make a direct comparison of the Syns in a well-described model of DAT trafficking. We have shown that while β-Syn and γ-Syn modulate NET trafficking, for example, this effect is not dependent on an intact microtubule cytoskeleton [22]. This distinguishes their mechanism of NET modulation from that employed by α-Syn, and suggests that the Syns work by multiple distinct pathways to influence pre-synaptic MAT function. To address remaining questions related to MAT trafficking, as well as to gain a deeper understanding of Syn function, we sought to analyze the mechanisms underlying DAT trafficking by the three Syns. We demonstrate for the first time that both β-Syn and γ-Syn can modulate DAT trafficking in a manner that is subtly different from α-Syn. Moreover, we demonstrate a novel process directing DAT cellular distribution that relies on modulation of export from the endoplasmic reticulum (ER) wherein altered trafficking of DAT by the various Syns is associated with attenuation of ER-Golgi transport and accumulation of DAT in the ER.

## Materials and Methods

### Ethics Statement

All studies with animals were approved by the Georgetown University Institutional Animal Care and Use Committee (Protocol 10–076).

### Reagents

Specific antibodies used for immunoblots (IB), immunocytochemistry (ICC), immunohistochemistry (IHC), or immunoprecipitation (IP) are listed in Table S1 in File S1. Radio-labeled dopamine ([^3^H]-DA, NET131, 24 Ci/mmol) was purchased from Perkin Elmer (Waltham, MA). All other reagents, except where indicated, were purchased from Sigma-Aldrich (St. Louis, MO).

### Cell culture and transfections

Human neuroblastoma SH-SY5Y cells were cultured in DMEM/F-12 50/50 media (Mediatech, Manassas, VA) as described [23]. All transfections used Fugene HD (Roche, Indianapolis, IN), as described by the manufacturer, 18 h after plating at a density of 10,000–30,000 cells per cm^2^ in various culture vessels (50–60% confluency). Plasmids used for transfection are listed in Table S2 in File S1.

### Dopamine uptake assay

[^3^H]-DA uptake assays were performed on adherent SH-SY5Y cells 48 h after transfection as described previously [23], [35]. Uptake of 20 nM [^3^H]-DA in Kreb's Ringer HEPES buffer (130 mM NaCl; 1.3 mM KCl; 2.2 mM CaCl_2_; 1.2 mM MgSO_4_; 1.2 mM KH_2_PO_4_; 10 mM HEPES; 1.88 g/L glucose; 100 μM ascorbic acid; 100 μM pargyline; pH 7.3) proceeded for 10 min and was terminated by washes with ice cold uptake buffer. Cells were homogenized and [^3^H]-DA uptake was measured by scintillation counter (Beckman). Nonspecific uptake was determined with the addition of 10 μM indatraline HCl.

### Cell viability assay

Cell viability was measured by MTT assay according to manufacturer's instructions (ATCC, Manassas, VA). Briefly, SH-SY5Y cells cultured and transfected as described above in 96 well plates were incubated for 48 h, the yellow tetrazolium MTT (3-(4, 5-dimethylthiazolyl-2)-2, 5-diphenyltetrazolium bromide) was added, then cells were incubated with MTT reagent for an additional 2 h until formazan salt precipitate formed. Cells and precipitate were solubilized, then dissolved formazan salt was quantified by absorbance at 570 nm on a plate reader (Biotek).

### Protein extraction, SDS-PAGE and immunoblot analysis

Homogenate (prepared in 10 mM Tris HCl, 100 mM NaCl, 1 mM EDTA, 1 mM EGTA, 250 mM sucrose, pH 7.4) protein concentration was determined by Lowry assay (Biorad), adjusted, and protein was extracted with the addition of detergents (1% Triton X-100, 0.5% Na-deoxycholate, 0.1% SDS) for 1 h at 4°C. Immunoblotting was performed as described previously [22] using 4–12% Bis-Tris NuPAGE gels (Invitrogen) and PVDF membranes (Biorad).

### Biotinylation of cell-surface proteins

Cell surface proteins in SH-SY5Y cells were biotinylated with sulfo-NHS biotin (1.0 mg/mL, Pierce) 24 h after transfection as described [36]. Briefly, cells were washed in PBS with 0.1 mM Ca^2+^ and 1.0 mM Mg^2+^ then incubated with sulfo-NHS biotin (1.0 mg/mL, Pierce) for 30 min at 4°C. Biotinylation was terminated by washing with 100 mM glycine and cell pellets were collected in PBS for lysis (see above). 300 μL of cleared total lysate (0.1 mg/mL protein) was incubated with 300 μL of streptavidin agarose beads (Pierce) for one h at room temperature then washed once in lysis buffer, twice in a high-salt buffer (500 mM NaCl; 50 mM Tris HCl; 5 mM EDTA; 0.1% Triton X-100; pH 7.5), and once in a no-salt buffer (50 mM Tris HCl; pH 7.5). Bound protein was eluted in 300 μL 2X sample buffer (120 mM Tris HCl; 0.02% bromophenol blue; 20% glycerol; 2% βME) as the biotinylated fraction. Total and biotinylated protein were analyzed simultaneously by SDS-PAGE.

### Confocal microscopy

All imaging was performed within the Georgetown University Medical Center (GUMC) Lombardi Comprehensive Cancer Center Microscopy and Imaging Shared Resource (LCC-MISR) on a Zeiss LSM 510 Meta confocal laser scanning microscope equipped with an argon laser producing excitation at 488 nm, helium-neon lasers producing excitation at 543 nm and 633 nm, and a Coherent Chameleon XR Ti:Sapphire laser producing two-photon excitation at various wavelengths (Table S3 in File S1). Fixed cells, live cells, and tissue were imaged with a Plan-Apochromat 63× or Plan-Apochromat 100× oil immersion objectives with a numerical apertures of 1.4. Immersion medium for all imaging was Immersol 518 F (Zeiss). Unless otherwise indicated, all imaging was conducted at room temperature for fixed materials or at 37°C for live cells. Live cell imaging was performed on an enclosed, heated stage with humidity, temperature, and atmosphere (5% CO_2_) maintained by the Axiovision incubation control software (Zeiss). Fluorochromes and corresponding optical configurations are listed in Table S3 in File S1. All microscopy was conducted in facilities supported and maintained by the GUMC LCC-MISR (NIH grant P30-CA051008).

### Fluorescence recovery after photobleaching (FRAP) analysis

Live SH-SY5Y cells were grown on No. 1.5 cover glass and separately co-transfected with equal amounts of DAT-mCherry and α-Syn-GFP, β-Syn-GFP, or γ-Syn-GFP. 24 h after transfection cells were placed on the incubated stage of a Zeiss LSM 510 microscope with a 63×1.4 Oil DIC objective and DAT-mCherry mobility was analyzed by FRAP in Syn-positive and Syn-negative cells (see Table S3 in File S1 for additional details). Guide images were captured at a resolution of 256×256 pixels (pixel size  = 93 nm) with illumination at 488 nm and 543 nm (see Table S3 in File S1) with constant laser intensity, gain, and offset applied to each channel. A 120 pixel by 80 pixel ROI (11.2×7.4 μm, 77.8 μm^2^) was defined and three pre-bleach images were collected followed by bleaching of a 30 pixel by 15 pixel rectangle (2.8×1.4 μm, 3.92 μm^2^) for 35 iterations (102.39 μsec/pixel) at full 543 nm laser intensity until 60–80% of fluorescence intensity was eliminated (I_POST_). Recovery was monitored for a pre-determined interval (27 s) established by optimization experiments to allow FRAP to reach a plateau without excessive further bleaching (I∞). The ROI was imaged every 0.3 s (89 images over 27 s) until DAT recovery reached a plateau (I∞) and intensity measurements and FRAP calculations were made from bleach and reference areas using the kinetic analysis module of Zeiss AIM software. The immobile fraction of DAT was calculated from FRAP time series according to curve fits to a single exponential: I(t) = I_POST_–((I∞–I_POST_)*exp^(−t*K)^). Additional processing was performed in ImageJ and Adobe Photoshop for presentation.

### Immunohistochemistry

IHC of mouse brain sections was performed as previously described [37] with slight modifications. 4% paraformaldehyde (PFA) perfused brains were embedded in optimal cutting temperature medium (Tissue Tek) and 10 µm sagittal cryosections were cut ∼1.2 mm lateral of the midline. Sections were warmed to −20°C, de-hydrated in acetone for 5 min at −20°C, then rinsed twice in room temperature phosphate buffer solution (PBSo; 24 g/L NaH_2_PO_4_, 114 g/L Na_2_HPO_4_, pH 7.4). Sections were permeabilized with 0.3% Triton X-100 in PBSo (PBSo-T) then blocked in PBSo-T with 10% normal horse serum (S-2000, Vector Labs, Burlingame, CA). Blocked sections were incubated overnight at 4°C with specific antibodies in blocking buffer (Table S1 in File S1), which were detected by AlexaFluor secondary antibodies (1∶200; Invitrogen). Stained sections were covered with Fluoromount-G containing DAPI (Southern Biotech, Birmingham, AL), and sealed with cover glass and clear nail polish.

### Immunocytochemistry

ICC of SH-SY5Y cells was performed as described previously with slight modifications [38]. Cells grown on culture slides (BD Biosciences, San Jose, CA) were fixed with 4% PFA for 30 min at room temperature, washed, and permeabilized with 0.3% Triton X-100 in PBSo (PBSo-T). Slides were blocked for one h in PBSo-T with 5% normal horse serum (S-2000, Vector Labs) then incubated overnight at 4°C with specific antibodies in blocking buffer (Table S1 in File S1), which were detected by AlexaFluor 568 and AlexaFluor 633 secondaries (1∶200; Invitrogen). Slides were covered with Fluoromount-G containing DAPI (Southern Biotech) and sealed with cover glass and clear nail polish.

### Endoplasmic reticulum to Golgi transport assay

SH-SY5Y cells grown to 50% confluence on culture slides (BD Biosciences, San Jose, CA) were transfected with 100 ng/cm^2^ tsVSVG-GFP (temperature sensitive vesicular stomatitis virus glycoprotein with green fluoresecent protein tag) and 400 ng/cm^2^ of α-Syn, β-Syn, γ-Syn, or empty vector and incubated for 24 h at 40°C. For 0 min time points, media was aspirated, cells were washed, and 4% paraformaldehyde was added immediately upon removal from the 40°C incubator. For later time points, culture slides were transferred to a 32°C incubator, kept there for designated intervals and then fixed. Fixed cells were processed for ICC of the Syn proteins and Gpp130 (Golgi phosphoprotein of 130 kDa), then images were captured and analyzed as described previously [39]. Confocal imaging parameters that could be applied to the majority of cells were defined empirically for each Syn protein, Gpp130, tsVSVG-GFP, and DAPI-stained nuclei. Cells were selected from random fields based on morphology and expression intensity of the Syn proteins (highest 25%) and tsVSVG-GFP (within analyzable dynamic range), then 12-bit confocal image stacks were captured in four optical planes spaced 0.7 μm apart. For image analysis, maximum projections were created in ImageJ for each channel and Golgi pixels were identified from the Gpp130 image (threshold  = 1000/4095). Using the Golgi pixels as a mask, mean tsVSVG-GFP intensity in the Golgi (V_G_) was calculated, then mean ER tsVSVG-GFP intensity (V_ER_) was estimated using a peripheral area defined by manually drawing an oval region of interest (ROI) between the nucleus (as labeled by DAPI) and the cell periphery. These ROI were placed on the side of the away from the cis-Golgi as identified by Gpp130 staining. Transport index (TI) was calculated for each measured cell separately as TI  =  V_G/_V_ER_, then means were determined for cells (20–30 total per group per time point) from three independent experiments.

### Subcellular fractionation

Separation of SH-SY5Y cells into cytosolic, membrane, and nuclear fractions was performed with a series of detergent extractions and centrifugations [40]. Cell pellets were re-suspended in buffer (150 mM NaCl; 50 mM HEPES) and permeabilized with 25 μg/mL digitonin for 10 min at 4°C to release cytosolic protein. Non-cytosolic materials were isolated by centrifugation at 2000 RCF for 10 min, cytosolic supernatant was collected, and the pellet was re-suspended in buffer with 1% NP40 to extract membrane proteins for 30 min at 4°C. Non-membrane materials were isolated by centrifugation at 7000 RCF for 10, membrane supernatant was collected, and the pellet was re-suspended in buffer with 0.5% sodium deoxycholate and 1% SDS to solubilize nuclear protein for 1 hour at 4°C. Each fraction was extracted in an equal volume of buffer then prepared for immunoblot analysis (see also Fig. S5A).

### Isolation of rough ER microsomes

Purified rough ER microsome fractions (ER/M) were isolated using the Sigma ER fractionation kit CaCl precipitation protocol as described previously [41]. 5×10^6^ cells per condition were pelleted at 600 RCF for 5 min at 4°C, then lysed in 1 mL hypotonic extraction buffer for 10 min at 4°C (10 mM HEPES; 1 mM EGTA; 25 mM KCl; pH 7.8). Lysed cells were centrifuged again at 600 RCF for 5 min at 4°C, then homogenized in 1 mL isotonic extraction buffer (250 mM sucrose; 10 mM HEPES; 1 mM EGTA; 25 mM KCl; pH 7.8) in a Dounce tube (30 strokes on ice). Homogenate was cleared by centrifugation at 1000 RCF for 10 min at 4°C (P2), and post-nuclear supernatant was cleared of mitochondria by centrifugation at 12000 RCF for 15 min at 4°C (P3). A CaCl solution (7.5 volumes) was added dropwise to the post-mitochondrial supernatant (microsome fraction) for a final concentration of 8 mM CaCl, with continuous agitation, then rotated for an additional 15 min before centrifugation at 30,000 RCF for 30 min at 4°C. ER/M pellets (P4) were re-suspended in isotonic extraction buffer (250 mM sucrose; 10 mM HEPES; 1 mM EGTA; 25 mM KCl; pH 7.8) containing 1% Triton X-100, 0.5% sodium deoxycholate, and 0.1% SDS then prepared for immunoblot analysis (see also Fig. S5C).

### Data analysis

Results are expressed as mean ± standard error of the mean (SEM) unless stated otherwise. ICA results were analyzed by a nonparametric sign-tests for values differing significantly from zero, as well as one-sample t-tests comparing means to zero [42], [43]. Results presented as averaged percent of control from multiple assays were analyzed by one-sample t-tests comparing each treatment to a theoretical mean of 100%, with a correction applied for multiple t-tests [44]. Results presented as means without standardization to percent of control were analyzed by one-way ANOVA with Dunnett's post-hoc analysis for statistically significant differences from control conditions. Statistical significance was accepted at p<0.05 and denoted with a single asterisk (*). Additional statistical distinctions were made at p<0.01 (**) and p<0.001 (***).

## Results

### Function and cell-surface distribution of DAT is reduced by synucleins

**Figure 1:**
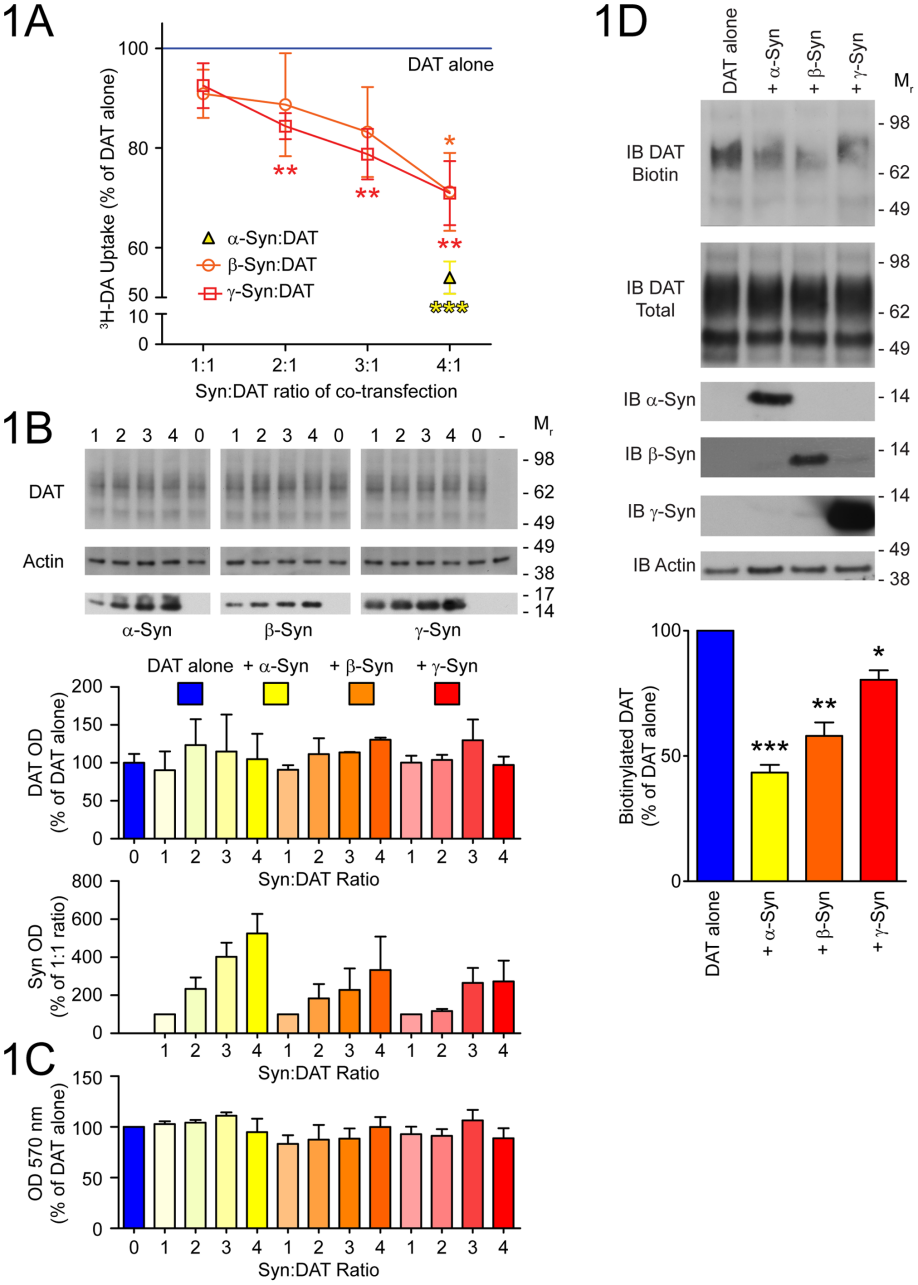
Function and cell-surface expression of DAT is reduced by synucleins. (A) Uptake of [^3^H]-DA into SH-SY5Y cells co-transfected with DAT (100 ng/cm^2^) and increasing amounts (100–400 ng/cm^2^) of β-Syn (orange open circles) or γ-Syn (red open squares) was measured over 10 min. Values (n = 6; mean ± SEM) are presented as percent of DAT alone (line at 100%). Non-specific uptake in the presence of 10 μM indatraline was subtracted. As a positive control for Syn modulation of DAT, uptake was also measured in cells co-transfected with DAT and 400 ng/cm^2^ of α-Syn (yellow triangle). Total amount of transfected DNA was kept constant at 500 ng/cm^2^ with the addition of empty pcDNA3.1 vector. (B) Expression levels of α-Syn, β-Syn, γ-Syn, and DAT in cells used for uptake assays were confirmed by immunoblot (IB). Representative blot images are shown and quantified relative to actin in the adjacent graphs (n = 3; mean ± SEM). (C) Viability was assessed by MTT assay in SH-SY5Y cells transfected under conditions identical to the uptake experiment. Results from three independent experiments assayed in quadruplicate are expressed as absorbance (OD) at 570 nm ± SEM. (D) Cell surface protein was biotinylated in SH-SY5Y cells co-transfected at the 4∶1 ratio as above. Biotinylated DAT (IB DAT Biotin) captured with streptavidin beads and total DAT (IB DAT Total) were measured by immunoblot. DAT biotinylation was quantified as optical density (OD) of DAT Biotin divided by OD of DAT Total relative to actin (n = 4; mean ± SEM). Molecular mass (M_r_) of nearest protein ladder bands is indicated. Data in were analyzed by t-test for difference from a theoretical mean of 100 (*p<0.05, **p<0.01, ***p<0.001) and corrected for multiple t-tests [44].

Accumulating evidence indicates that the neurochemical and behavioral effects of the loss of α-Syn are either partially [32] or totally [14], [33], [45] masked *in vivo*. Our own analysis echoes these reports, showing that deletion of α-Syn has no effect on DAT distribution or DA-related behaviors (see Fig. S1). These prior findings raise the possibility that a compensatory response occurs in these animals, potentially due to redundancy between the Syn proteins. [^3^H]-DA uptake by DAT is dependent on localization of the transporter to the plasma membrane and is reduced by α-Syn trafficking of DAT away from the cell surface and into the cytoplasm [28], [29], [46], [47], [48]. To assess whether β-Syn and γ-Syn can also modulate DAT function, [^3^H]-DA uptake by DAT was examined in SH-SY5Y cells co-transfected to express a constant amount of DAT and increasing concentrations of β-Syn or γ-Syn relative to DAT (1–4 μg Syn plasmid/1 μg DAT plasmid). α-Syn was also co-expressed at a 4∶1 ratio (α-Syn:DAT) as a positive control for DAT modulation (Fig. 1A). Ratios of Syn:DAT ≥2∶1 caused progressive reductions in the uptake of [^3^H]-DA compared to cells expressing DAT alone, and at a 4∶1 ratio of any Syn to DAT, statistically significant reductions in [^3^H]-DA uptake were detected (Fig. 1A). Increasing Syn:DAT co-transfection ratios led to increased levels of the Syns but no changes in total DAT expression (Fig. 1B), confirming that decreased [^3^H]-DA uptake was not due to decreased DAT expression. Co-transfection at the 4∶1 ratio with DAT resulted in Syn levels that were at or slightly above those in striatal tissue (Fig. S2A). Nonetheless, this level of expression still under-estimated by a significant margin the Syn:DAT ratio present in the striatum (see Fig. S2B). Thus, Syn over-expression in transfected SH-SY5Y cells produced a physiologically relevant Syn:DAT ratio [49]. Over-expression of the Syns at these levels did not cause changes in cell viability compared to cells expressing DAT alone (Fig. 1C) or other toxic effects. For example, proteasome activity, a cellular function known to be impaired by extreme over-expression of the Syns [50], [51], is unaltered in Syn transfected cells (Fig. S2B–S2C).

To determine whether the reduced uptake of [^3^H]-DA by DAT in the presence of increased levels of the Syns was associated with decreased cell-surface distribution of DAT, biotinylation of DAT in intact co-transfected SH-SY5Y cells was conducted (Fig. 1D). Indeed, levels of biotinylated DAT were significantly reduced upon co-transfection with α-Syn (43±6%, p<0.001), β-Syn (58±11%, p<0.01) and γ-Syn (80±8%, p<0.05). The decrease in biotinylated DAT was not due to changes in DAT expression, as similar levels of total DAT protein were present in all conditions (Fig. 1D). Interestingly, these experiments revealed that a significant portion of DAT is retained in intracellular compartments, even in the absence of the Syn proteins, as only a fraction of total DAT is recovered by biotinylation (Fig. S3A). Quantification of biotinylated DAT in the presence or absence of permeabilizing detergent showed that approximately 50% of total DAT is on the cell surface (Fig. S3B), consistent with previous reports [31], [52].

### Synucleins limit the immobile fraction of DAT

We next examined Syn modulation of DAT trafficking using FRAP (fluorescence recovery after photobleaching) analysis conducted in live SH-SY5Y cells. Monitoring of FRAP allows the separate quantification of the freely diffusing intracellular population of DAT (mobile fraction) and the relatively static plasma membrane resident population of DAT (immobile fraction). Insertion and diffusion of plasma membrane proteins is typically slower than the rate of intracellular diffusion and therefore the plasma membrane population is not recovered over the time scale of these FRAP experiments. For example, diffusion of DAT in the plasma membrane has been estimated by FRAP at between 0.01 and 0.03 μm^2^/s [53], a rate that is more than 10-fold slower than similar proteins in intracellular compartments [54]. Thus, measurement of the portion of initial fluorescence (I_PRE_) that is not recovered following photobleaching (FI, the immobile fraction) provides a real-time estimate of the relative size of the cell surface population of the monitored protein [55].

**Figure 2:**
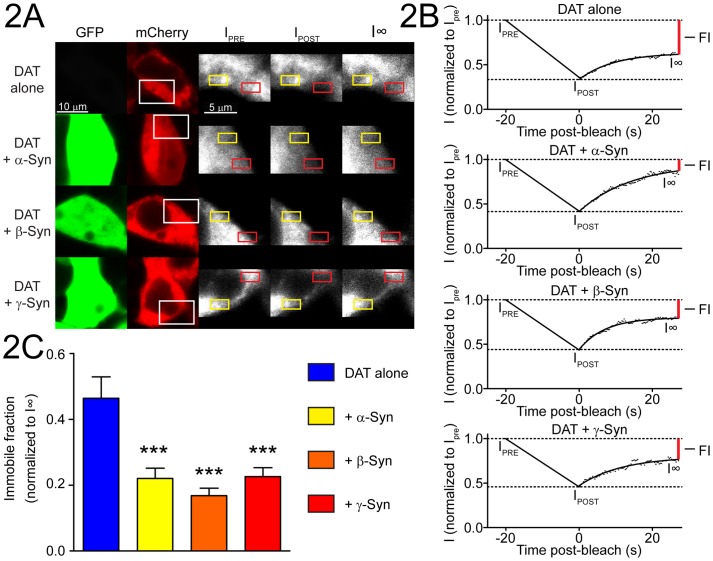
Synucleins limit the immobile fraction of DAT. SH-SY5Y cells were transfected with DAT-mCherry and α-Syn-GFP, β-Syn-GFP, or γ-Syn-GFP and DAT-mCherry mobility was analyzed by FRAP in live Syn-positive and Syn-negative (DAT alone) cells. (A) Guide images were captured (GFP and mCherry columns) and a region of interest (120×80 pixels) was defined (white rectangles) and three pre-bleach images (I_PRE_) were collected followed by bleaching a 30×15 pixel area (red rectangles) so that between 20% and 40% of DAT signal was retained (I_POST_). Representative confocal images of photo-bleached cells (I_POST_) are shown with bleached areas and corresponding reference areas indicated (yellow rectangles), with recovery shown at 27 s post-bleach (I∞). (B) Representative recovery curve fits of FRAP data from DAT-alone cells or DAT-mCherry co-transfected with α-Syn-GFP, β-Syn-GFP, or γ-Syn-GFP. Pre-bleach (I_PRE_) and post-bleach (I_POST_) intensities are indicated with dashed lines. Recovery curves normalized to I_PRE_ are fit to background- and bleach-corrected DAT-mCherry intensity at each time point, with plateau indicated at I∞. The immobile fraction of DAT (FI; I_PRE_ - I∞) in representative curves is indicated by vertical red lines at t = 27 s, and was calculated from a single exponential curve fit: I(t) = I_POST_–((I∞–I_POST_)*exp^(−t*K)^). (C) FI data were compiled from three experiments conducted independently on five cells per condition (n = 15; mean ± SEM; see also Table 1). FI data were analyzed by one-way ANOVA followed by Dunnet's post-hoc analysis for comparison to DAT-alone cells (***p<0.001).

SH-SY5Y cells growing on cover slips were co-transfected with DAT-mCherry and GFP-tagged α-Syn, β-Syn, or γ-Syn, and DAT-mCherry recovery was monitored by FRAP in both GFP-postiive and GFP-negative cells (Fig. 2A). An area near the periphery was selected in cells with moderate levels of DAT-mCherry expression (Fig. 2A; white boxes). Within this field, two regions of interest were defined, one for bleaching (Fig. 2A; red boxes) and one for an internal reference (Fig. 2A; yellow boxes). Upon bleaching (I_POST_), fluorescence intensity was reduced to 20–40% of initial values (I_PRE_). Following bleaching, DAT-mCherry intensity recovered to approximately 55% of I_PRE_ in GFP-negative cells (DAT alone; Fig. 2B; see Video S1), leaving an immobile fraction of 45%. A representative recovery curve normalized to I_PRE_ is shown with the immobile fraction indicated (FI; Fig. 2B; vertical red lines). In the presence of the Syn-GFP proteins, FI was decreased, with DAT-mCherry intensity recovering a larger fraction of initial fluorescence. The timing of recovery was similar under all conditions, and no significant differences were detected in K of DAT-mCherry FRAP (Fig. 2B; Table 1). This kinetic analysis indicates that the FI of DAT-mCherry was significantly decreased in cells expressing α-Syn-GFP, β-Syn-GFP, or γ-Syn-GFP (Fig. 2C and Table 1; ***p<0.001). Taken as a measure of plasma membrane resident DAT, the decrease in FI suggests that the fraction of DAT in the extracellular membrane was reduced in the presence of the Syn proteins and is consistent with reduced [^3^H]-DA uptake and cell-surface occupancy of DAT.

**Table 1:** Kinetic analysis of DAT-mCherry FRAP^a^.

Co-transfection	*n*	K (1/s)^b^	Mobile fraction (FM)^c^	Immobile fraction (FI)^d^
DAT alone	15	0.095±0.007	0.454±0.021	0.464±0.065
α-Syn + DAT	15	0.104±0.009	0.398±0.024	0.220±0.031 ***
β-Syn + DAT	15	0.096±0.011	0.450±0.031	0.168±0.023 ***
γ-Syn + DAT	15	0.116±0.015	0.431±0.031	0.226±0.027 ***

a Performed on transfected SH-SY5Y cells as described (Materials and methods; Fig. 2) with Kinetic Analysis module of Zeiss AIM software. ^b^K of fluorescence recovery (presented as mean ± SEM) calculated for a single exponential from background corrected fluorescence intensity at each time (I(t)) according to: [I(t) = I_POST_–((I∞–I_POST_)*exp^(−t*K)^)] where I_POST_  =  fluorescence intensity immediately following bleaching period and I∞  =  plateau of fitted recovery curve (I∞ normalized to 1 for calculations). ^c^Mobile fraction of DAT-mCherry (FM) determined according to FM  =  I∞ – I_POST_. ^d^Immobile fraction of DAT-mCherry (FI) determined according to FI  =  I_PRE_ – I∞, where I_PRE_  =  fluorescence intensity immediately prior to initiation of bleaching. Data are compiled from three experiments conducted independently on five cells per condition (n = 15) and are presented as mean ± SEM and analyzed by one-way ANOVA followed by Dunnet's post-hoc analysis for difference from DAT alone control (*** = p<0.001).

### Co-localization of synucleins with DAT

**Figure 3:**
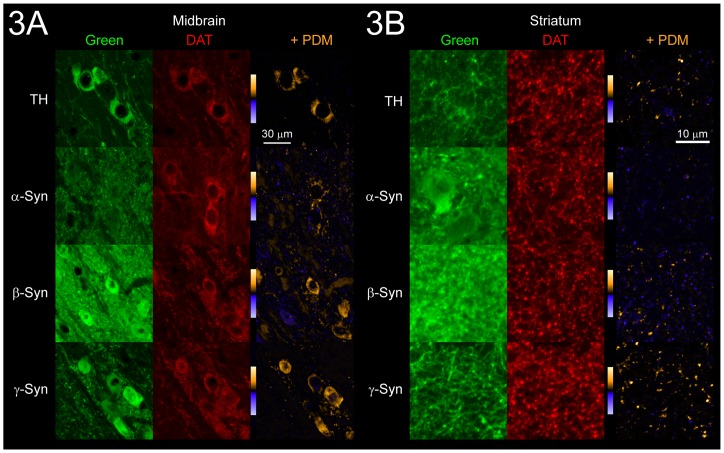
Synucleins and DAT in the brain. Co-distribution of TH, α-Syn, β-Syn, and γ-Syn with DAT was assessed by intensity correlation analysis (ICA) in (A) midbrain and (B) striatal tissue co-stained with each Syn-DAT pair (or TH reference). Representative images from each tissue of the Syn-DAT pairs are shown with Syn proteins or TH (green) in the first column, corresponding images of DAT (red) in the second column, and PDM (product of the difference of the means) images generated from ICA in the third column (+ PDM; see also Supporting information in File S1, Fig. S4B-S4C, and Table S4 in File S1). Color scales are embedded in PDM images where yellow represents areas of positive co-variance and blue represents areas of negative co-variance.

Syns are known to be present in the striatum and the midbrain [56], and we have shown that the Syn:DAT ratio in the striatum was at or above the ratio present in co-transfected cells (Fig. S2B). To determine whether the Syns and DAT are co-distributed to the same cells *in vivo*, sagittal brain slices from WT mice containing both the substantia nigra (SN) and striatal regions were immunostained for the Syns and for DAT. Tyrosine hydroxylase (TH) was also visualized as a reference for a protein known to be co-expressed with DAT. Images showed close overlap between TH and DAT in SN cell bodies found in the midbrain (Fig. 3A). Overlap was also observed between the Syns and DAT, though expression of α-Syn and β-Syn was widespread throughout the midbrain (Fig. 3A). γ-Syn expression was largely restricted to DAT-expressing SN cells (Fig. 3A). Overlap between TH and DAT was similarly high in striatal regions, as expected (Fig. 3B). Though α-Syn and β-Syn were co-distributed with DAT to some extent, expression of both was high throughout the striatum, including areas where DAT was absent (Fig. 3B). Most interestingly, striatal γ-Syn expression was also restricted to DAT expressing cells (Fig. 3B). Indeed, γ-Syn, was co-expressed with DAT throughout the entire nigrostriatal pathway, including fibers connecting the midbrain and striatum (Fig. S4A). Intensity correlation analysis [43] confirms that γ-Syn is closely co-distributed with DAT in the midbrain and the striatum, while α-Syn and β-Syn are more widely dispersed (Fig. S4B-S4C and Table S4 in File S1). These combined data indicate that each of the Syns is differentially co-distributed with DAT throughout the nigrostriatal pathway, and suggest that localized concentrations of the Syns may be an important determinant of their regulation of DAT trafficking in the brain.

### Synucleins attenuate export from the endoplasmic reticulum to the Golgi

**Figure 4:**
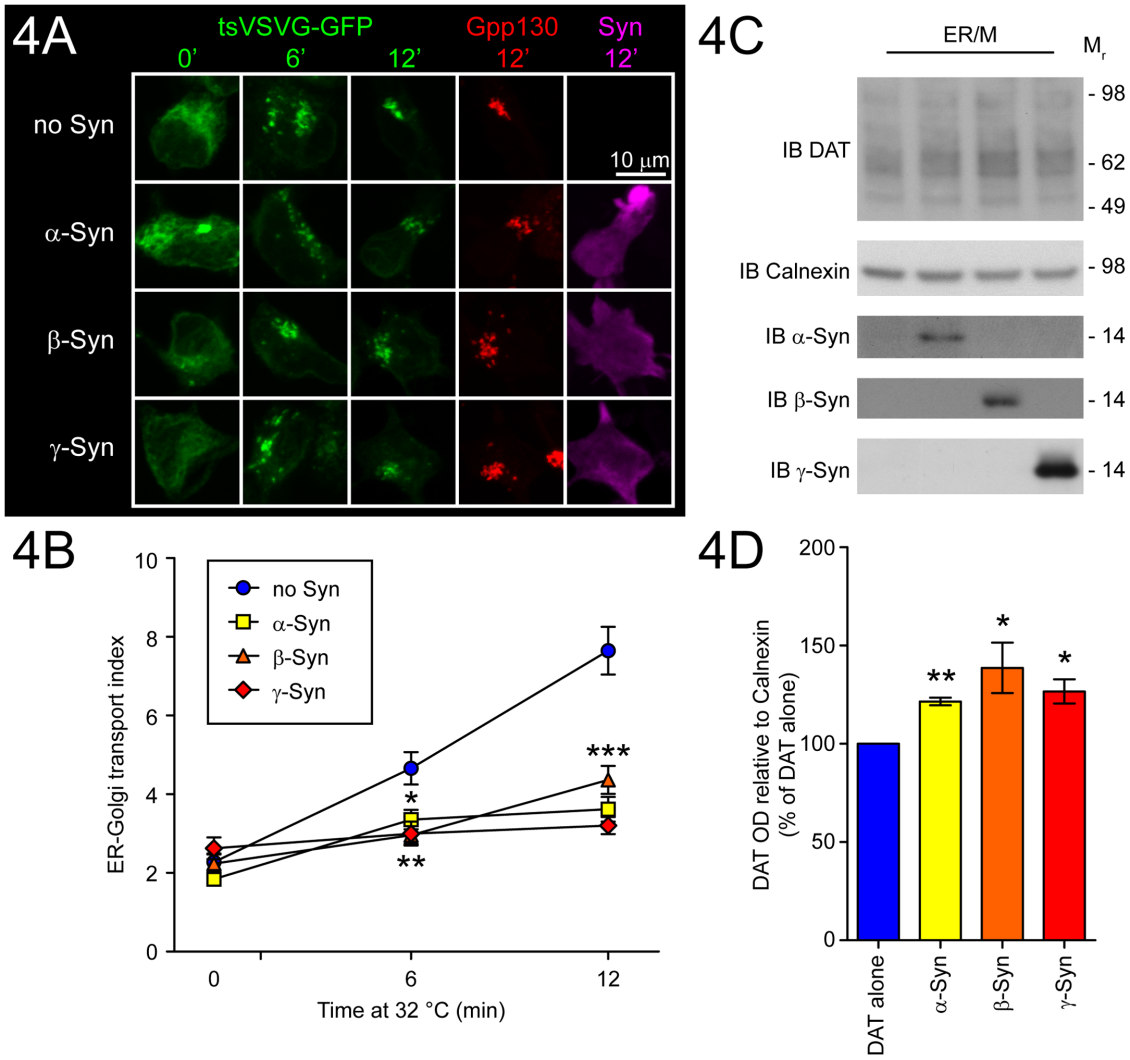
Attenuation of the ER-Golgi transition by α-Syn, β-Syn, and γ-Syn. The ER-Golgi transition was analyzed by the tsVSVG-GFP transport assay (see Materials and methods). (A) Representative tsVSVG-GFP images from 0, 6, and 12 min after temperature shift are shown for each condition. 12 min images are paired with Gpp130 images used to define Golgi area, and images of the respective stains for α-Syn, β-Syn, or γ-Syn. (B) Transport index was calculated from 5–10 cells per condition from three independent experiments (15–30 total cells per condition). Values are presented as mean ± SEM and are analyzed by one-way ANOVA with Dunnett's post-hoc analysis at each time point for comparison to DAT alone control (*p<0.05, **p<0.01, ***p<0.001). (C) ER microsomes (ER/M) were prepared from SH-SY5Y cells transfected with DAT (100 ng/cm^2^) and (400 ng/cm^2^) of α-Syn, β-Syn, or γ-Syn and analyzed by immunoblot for expression of DAT relative to Calnexin (loading control). (D) Data are presented as percent of DAT alone control (n = 3–4; mean ± SEM). Means were analyzed by one sample t-tests for difference from a theoretical mean of 100 (* = p<0.05, ** = p<0.01).

A significant fraction of total DAT is localized within intracellular compartments of transfected SH-SY5Y cells, and overlaps spatially with both ER and Golgi markers (see Videos S2–S4). Trafficking of DAT out of intracellular compartments and to the plasma membrane is partially dependent on ER-Golgi export, a process that is known to be influenced by α-Syn [39]. To determine whether Syns could impede ER-Golgi transport under the conditions where DAT trafficking occurred, a temperature sensitive mutant of VSVG-GFP (tsVSVG-GFP [57]) was used to assess the ER-Golgi transition in the presence or absence of the Syns. Although at 37°C VSVG-GFP was distributed to vesicles and the cell periphery (see Video S4), maintenance of SH-SY5Y cells at 40°C caused retention of tsVSVG-GFP in the ER (Fig. 4A; 0′) as described previously [58]. The distribution of tsVSVG-GFP was dramatically altered at this temperature, with almost no vesicles apparent (see Video S5). Release of tsVSVG-GFP from the ER was restored by returning cells to a lower temperature environment, resulting in depletion of tsVSVG-GFP signal from ER compartments and concentration into cis-Golgi vesicles (Video S5). The change in tsVSVG-GFP distribution following a shift in temperature from 40°C to 32°C was used to calculate the ER-Golgi transport index, a measure of the rate of the ER-Golgi transition [58], [59].

**Figure 5:**
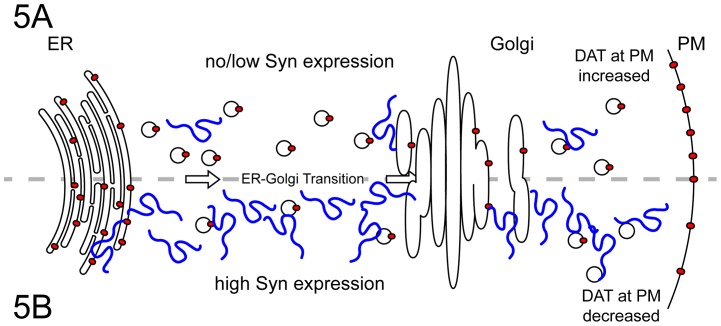
Contribution of synuclein-dependent attenuation of ER export to synuclein modulation of DAT. Under normal conditions (A) where the Syns are absent or at low levels, DAT (red ovals) export is maintained at normal levels, with a relatively increased amount of plasma membrane (PM) distribution. When Syn expression is elevated (B) ER export and the ER-Golgi transition are attenuated, leading to retention of DAT and other secreted proteins in the ER. Thus, although overall expression of the transporter remains unchanged, DAT distribution to the PM is decreased.

It has previously been shown that both wild-type and A53T α-Syn can delay the ER-Golgi transition of tsVSVG-GFP in PC-12 neuroendocrine cells [39]. However, the effects of β-Syn or γ-Syn on tsVSVG-GFP and the ER-Golgi transition are not known, nor have any studies, to our knowledge, examined Syn-dependent effects on tsVSVG-GFP in SH-SY5Y cells. Following co-transfection of SH-SY5Y cells with tsVSVG-GFP and the Syns, cells were maintained at 40°C for 24 h (Fig. 4A; 0′). Upon exposure of cells to 32°C, dispersed tsVSVG-GFP rapidly condensed into small vesicles, which became apparent within 6 min and were well formed at 12 min (Fig. 4A; 6′ and 12′). The tsVSVG-GFP vesicles occupied the peri-nuclear region, and the transition was complete within 15 to 20 min of the temperature shift (Video S5). By assessing the intensity of tsVSVG-GFP signal that overlapped with Gpp130 (Golgi phosphoprotein of 130 kDa), a marker of the cis-Golgi compartment, the ER-Golgi transport index was derived for each experimental condition at 0, 6, and 12 min after the temperature shift (Fig. 4B). Upon co-expression with Syns, the transition of tsVSVG-GFP from the ER to the cis-Golgi compartment was significantly delayed, even as early as 6 min with α-Syn (p<0.05), β-Syn and γ-Syn (p<0.01), with a larger difference observed at 12 min (p<0.001). This suggests that when present, the Syns may similarly decrease the rate of ER-Golgi transition of DAT, causing it to be abnormally retained in the ER. All three Syns were found in membrane fractions (see Fig. S5A–S5B) and were also associated with purified ER microsomes (Fig. 4C; see Fig. S5C for ER isolation). Consistent with an impairment of the ER-Golgi transition (Fig. 4A–4B), we observed accumulation of DAT within purified ER microsomes (Fig. 4C–4D), an indication that expression of the Syn proteins reduced trafficking of DAT out of the ER. Taken together, the Syn-dependent effects on the ER-Golgi transition and accumulation of DAT in the ER strongly support the conclusion that the Syns modulate DAT distribution in part through a generalized attenuation of ER export (Fig. 5A–5B).

## Discussion

Here, we have generated novel data demonstrating that modulation of DAT trafficking by the Syn family of proteins occurs through multiple, distinct mechanisms. Like α-Syn, transfection of either β-Syn or γ-Syn modulated distribution of DAT. Over-expression of any of the Syns was sufficient to reduce trafficking of DAT to the cell surface, producing decreased uptake of [^3^H]-DA by limiting the population of plasma membrane-resident DAT. Transport of model secretory protein VSVG out of the ER was significantly delayed upon co-transfection with any of the Syn proteins. This attenuation of the ER-Golgi transition was associated with reduced export of DAT from the ER and its accumulation in this compartment. Together, our findings contribute to an emerging consensus that the Syn proteins regulate biosynthetic processes [60].

Numerous studies have demonstrated a striking impairment of the ER-Golgi transition and vesicle trafficking by α-Syn, though these models frequently resulted in toxic effects [27], [61], [62]. Perhaps even more intriguing are models where Syn-dependent effects on ER-Golgi function and vesicle trafficking were evident, but these and other vital cell functions appeared largely intact, suggesting a regulatory role for the Syns [39], [63]. Synthesis and trafficking of DAT through the ER, Golgi, and post-Golgi compartments is subject to regulation at many stages and by a wide array of factors, including many protein binding partners [64], [65]. DAT contains 12 transmembrane domains that require co-translational translocation into the ER membrane, as well as three extracellular N-linked glycosylation sites that can influence DAT distribution [66]. Golgi function is also critical for normal trafficking of DAT; for example, disruption of Golgi integrity following proteasome inhibition causes intracellular accumulation of DAT [58]. Both the ER-Golgi transition and integrity of the Golgi apparatus are known to be sensitive to the over-expression of α-Syn [27], [61]. Recent reports show that α-Syn can enter the ER and interact with chaperones that are critical for normal ER function [67], [68], and this effect has also been reported in a mouse model of synucleinopathy [69], [70]. β-Syn and γ-Syn also appear competent to influence passage of DAT through these compartments, as they possess both lipid-binding [71] and chaperone-like properties [72]. Indeed, similar effects of all three Syns were observed on the ER-Golgi transition under our experimental conditions in SH-SY5Y cells, with the ER-Golgi transport index significantly reduced in the presence of α-Syn, β-Syn, or γ-Syn.

Once properly targeted to the synapse, regulated insertion of DAT into the plasma membrane is a critical mode of clearing released dopamine from the synaptic cleft, and is the only means for re-uptake and recycling of dopamine [64], [65]. Syntaxin-1A is a SNARE protein that interacts with and can facilitate membrane insertion of DAT [73]. Assembly of SNARE complexes that include Syntaxin-1A and other critical components of the exocytotic machinery are directly affected by availability of the Syn proteins, as deletion of all three endogenous Syn proteins in triple KO mice impairs SNARE complex formation [11], [74]. This deficit is rescued by exogenous re-expression of human α-Syn, which restores SNARE complex formation in neurons from these animals [11]. Importantly, it has also been demonstrated that over-expression of a truncated form of α-Syn in transgenic mice alters Syntaxin-1A and DAT distribution in the striatum [75]. These earlier findings illustrate that the Syns have the potential for involvement at nearly every stage of the DAT lifecycle and that expression either above or below an optimal threshold has direct consequences for fundamental neuroregulatory mechanisms. Our present data extend these consequences further upstream to DAT export from the ER, and are consistent with recent findings pointing to a role for all three Syns as cooperating co-regulators of synaptic function. Importantly, this mechanism for Syn-dependent modulation of DAT differs from a process we have described previously involving a stable protein-protein interaction and tethering of DAT to microtubules by α-Syn, which we were unable to confirm for β-Syn or γ-Syn (see Fig. S6). Thus, modulation of DAT by the Syns operates simultaneously through multiple mechanisms, including parallel effects on ER and Golgi function.

Taken together, our results demonstrate a potentially important role for all three Syns in affecting ER function, and show how this function contributes to modulation of DAT trafficking. Though prior studies showed that the NAC region of α-Syn mediates microtubule tethering of DAT to modulate DAT distribution, it is clear from the present work that modulation of DAT trafficking is not solely dependent on this interaction. A separate contributor to DAT trafficking is the indirect effect of α-Syn, β-Syn, and γ-Syn on export of ER-resident proteins to the Golgi. The existence of multiple layers of interlocking, redundant regulation DAT is consistent with overlapping roles for all three Syns. Thus, while α-Syn is often viewed as having a special relationship with dopaminergic neurotransmission, it is crucial to recognize now that β-Syn and γ-Syn are also present in the relevant tissues and are equally capable of significant regulation of DAT, a critical member of the dopamine signaling network.

## Supporting Information

Figure S1 **Neurochemical and behavioral status of α-Syn KO mice.** Striatal protein isolated from littermate WT and α-Syn KO mice was analyzed by immunoblot (A) as total lysates (TL) or membrane fractions (MEM) for expression levels of α-Syn, β-Syn, γ-Syn, and DAT. Actin expression was also analyzed as a loading control. Representative blot images from each genotype are presented with approximate molecular mass of nearest protein ladder bands indicated (M_r_). Band optical density (OD) relative to actin is presented as percent of WT (mean ± SEM). Comparisons between WT (n = 10) and α-Syn KO (n = 10) were made for each protein by t-test (*p<0.05). Open field (OFT), elevated plus maze (EPM), and forced swim tests (FST) were performed on WT (n = 12) and α-Syn KO (n = 12) mice to analyze motor activity, anxiety-like behavior, and depressive-like behavior. (B) Distance traveled and center zone entries on the OFT, and (C) open arm entries on EPM were measured by automated video tracking using ANY-maze software. Immobility score on the FST was determined from video recordings by a blinded observer counting the number of 5 s bins each animal spent in an immobile posture. Results are presented as mean ± SEM and were analyzed by t-test (no significant differences detected).
(TIF)

Figure S2 **Cellular conditions associated with over-expression of synucleins.** (A) Protein isolated from rat striatum and SH-SY5Y cells transfected with with 100 ng/cm^2^ DAT and 100–400 ng/cm^2^ of vector alone, α-Syn, β-Syn, or γ-Syn was analyzed by immunoblot. Protein loading was adjusted to generate equal immunodetection of DAT in each sample, and expression levels of α-Syn, β-Syn, and γ-Syn were compared between rat tissue and transfected cells. (B) Samples from A were adjusted for equal protein loading as indexed by actin immunoreactivity. Expression levels of α-Syn, β-Syn, and γ-Syn were compared between rat tissue and transfected cells. 10–240 pg/lane of purified recombinant α-Syn, β-Syn, or γ-Syn were immunoblotted simultaneously to estimate Syn abundance in each sample. (C) Expression levels of 19S proteasome subunit S6′ and 20S proteasome subunit α5 were assessed by immunoblot in cells transfected with 100 ng/cm^2^ DAT and 400 ng/cm^2^ of vector alone, α-Syn, β-Syn, or γ-Syn (n = 3). Syns and DAT were also probed to verify experimental conditions. GAPDH is displayed as a control for protein loading. Representative blot images from each condition are presented with approximate molecular mass of nearest protein ladder bands indicated (Mr). (C) Lactasystin-sensitive digestion of the model substrate suc-LLVYAMC was used to measure 26S proteasome activity in cells transfected as above. Proteasome activity is expressed as mean fluorescence ± SEM (excitation 355 nm; emission 460 nm). Data were analyzed by one-way ANOVA followed by Dunnet's post-hoc analysis for comparison to DAT-alone cells (n = 4; no significant differences detected).
(TIF)

Figure S3 **Distribution of DAT in transfected SH-SY5Y cells.** (A) Direct comparison by immunoblot of DAT Total and DAT Biotin from cell surface biotinylation experiments (see also Fig. 1D). (B) Recovery of biotinylated DAT from intact or Triton X-100 permeabilized cells as quantified by o-phenyldiamine absorbance (see Supporting methods in File S1).
(TIF)

Figure S4 **Synucleins and DAT in the brain.** (A) Co-labeling of γ-Syn (green) and DAT (red) in dopaminergic fibers of the nigrostriatal pathway. Close correspondence of γ-Syn with DAT-positive structures is shown in the merge. Co-distribution of TH, α-Syn, β-Syn, and γ-Syn with DAT was assessed by intensity correlation analysis (ICA) in immunostained midbrain and striatal tissue (see Fig. 3A–3B for images). All Syn proteins and TH were positively co-distributed with DAT in these tissues (see Table S5 in File S1). Box-and-whisker plots (whiskers, 2.5^th^ and 97.5^th^ percentiles, boxes, 25^th^, 50^th^, and 75^th^ percentiles) display ICQ values from (B) midbrain and (C) striatal fields analyzed for each pair. One-way ANOVA with Dunnett's post-hoc analysis was also used to compare Syn-DAT ICQ means to TH-DAT (***p<0.001).
(TIF)

Figure S5 **Cell fractionation and ER/M isolation controls**. (A) SH-SY5Y cells were transfected with DAT (100 ng/cm^2^) and (400 ng/cm^2^) of α-Syn and cytosol, membrane, and nuclear fractions were prepared. Fractions were analyzed by immunoblot for expression of DAT, α-Syn, and markers for cytosol (glyceraldehyde 3-phosphate dehydrogenase, GAPDH), membrane (Grp78, Na/K ATPase, and voltage dependent ion channel, VDAC), and nuclear fractions (Lamin B). (B) Cytosol and membrane preparations from cells transfected with DAT (100 ng/cm^2^) and (400 ng/cm^2^) of empty vector, α-Syn, β-Syn, or γ-Syn were analyzed by immunoblot for expression of the Syns. (C) SH-SY5Y cells were harvested and processed for isolation of purified rough ER/M. Pellets (P2, P3, P4; see Materials and methods) were extracted in buffer containing detergents and analyzed by immunoblot for expression of markers for nuclear material (Lamin B), plasma membrane (Na/K ATPase), mitochondria (VDAC), and ER (Calnexin and Grp78).
(TIF)

Figure S6 **Analysis of interactions between synucleins and DAT.** (A) SH-SY5Y cells were co-transfected with DAT and α-Syn, β-Syn, or γ-Syn and lysed for immunoprecipitation (IP) with antibodies (Table S1 in File S1) against Syns or DAT. IP from identical samples using pre-immune IgG (sheep, Shp IgG IP; rabbit, Rbt IgG IP) was performed in parallel as a control for specificity of each IP and co-IP. Input (5%) and IPs were analyzed by immunoblots probed (IB) for Syns and DAT. Blot images are representative of three independent experiments; faint non-specific bands were observed below 14 kDa on blots probed for β-Syn. (B) Uptake of [^3^H]-DA SH-SY5Y cells transfected with DAT (100 ng/cm^2^) and (400 ng/cm^2^) of empty vector, β-Syn, or γ-Syn was measured following treatment with 10 μM nocodazole (NOC) as described previously [12]. Values recorded from three assays performed in triplicate (mean ± SEM) are presented as percent of DAT alone (line at 100%). Non-specific uptake in the presence of 10 μM indatraline was subtracted. Uptake data were analyzed by t-test for difference from a theoretical mean of 100 (*p<0.05) and corrected for multiple t-tests [20].
(TIF)

File S1 **Additional supporting information, including Supporting methods, results, tables, and references.**
(DOC)

Video S1 **DAT-mCherry FRAP time series.** Representative FRAP time series of DAT-mCherry expressed in SH-SY5Y cells alone or in the presence of α-Syn-GFP, β-Syn-GFP, or γ-Syn-GFP. Three pre-bleach frames are shown, followed by the 27 s recovery period after photobleaching. Images presented in Fig. 2 are taken from the time series shown here.
(AVI)

Video S2 **DAT-mCherry with ER-TrackerTM time series.** Time series captured over approximately 18 min through a single optical section of SH-SY5Y cells expressing DAT-mCherry and stained with the ER-TrackerTM dye.
(AVI)

Video S3 **DAT-mCherry with ER-TrackerTM z-stack time series.** Time series captured over approximately 8 min through six adjacent optical sections (0.80 μm apart) of SH-SY5Y cells expressing DAT-mCherry and stained with the ER-TrackerTM dye. Z-stack time series loops continuously and is rotated along the x-axis and then the y-axis for display purposes.
(AVI)

Video S4 **DAT-mCherry with VSVG-GFP time series.** Time series captured over approximately 7 min through a single optical section of SH-SY5Y cells expressing DAT-mCherry and VSVG-GFP. White arrow that appears from 00∶47 to 03∶18 indicates a large moving vesicle that contains both DAT-mCherry and VSVG-GFP fluorescence.
(AVI)

Video S5 **tsVSVG-GFP temperature shift time series.** Time series captured over approximately 20 min through a single optical section of SH-SY5Y cells expressing VSVG-GFP that have been maintained at 40°C for 24 h following transfection. Temperature is lowered from 40°C to 32°C over 5 min, with the last 15 min of the time series captured at the lower temperature.
(AVI)
