# Supplementary figures and images for: Synucleins Antagonize Endoplasmic Reticulum Function to Modulate Dopamine Transporter Trafficking
Source: PLoS One. 2013 Aug 13;8(8):e70872. doi: 10.1371/journal.pone.0070872 (PMC3742698; doi:10.1371/journal.pone.0070872)

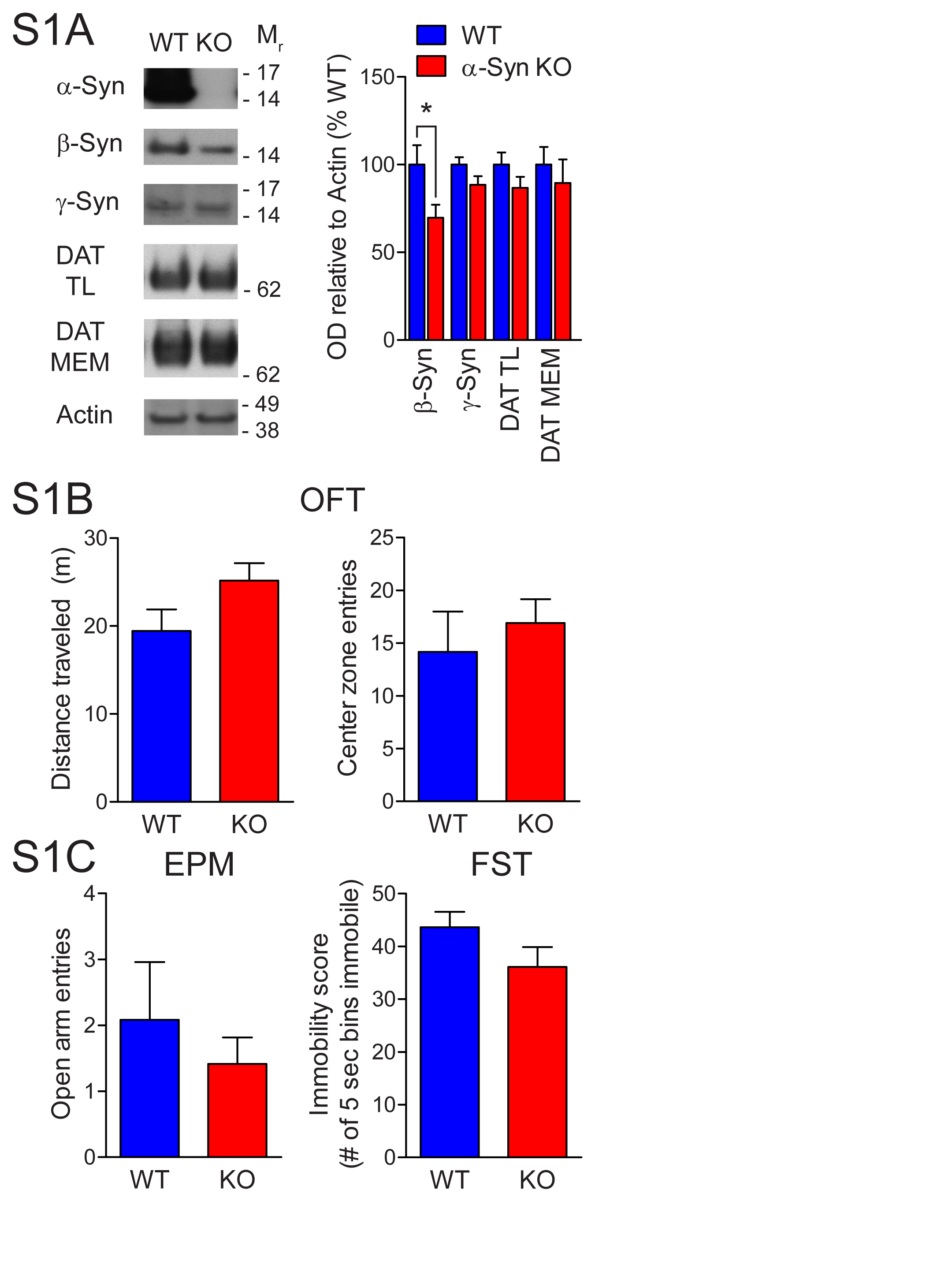

Supplement: Figure S1 — Neurochemical and behavioral status of α-Syn KO mice. Striatal protein isolated from littermate WT and α-Syn KO mice was analyzed by immunoblot (A) as total lysates (TL) or membrane fractions (MEM) for expression levels of α-Syn, β-Syn, γ-Syn, and DAT. Actin expression was also analyzed as a loading control. Representative blot images from each genotype are presented with approximate molecular mass of nearest protein ladder bands indicated (Mr). Band optical density (OD) relative to actin is presented as percent of WT (mean ± SEM). Comparisons between WT (n = 10) and α-Syn KO (n = 10) were made for each protein by t-test (*p<0.05). Open field (OFT), elevated plus maze (EPM), and forced swim tests (FST) were performed on WT (n = 12) and α-Syn KO (n = 12) mice to analyze motor activity, anxiety-like behavior, and depressive-like behavior. (B) Distance traveled and center zone entries on the OFT, and (C) open arm entries on EPM were measured by automated video tracking using ANY-maze software. Immobility score on the FST was determined from video recordings by a blinded observer counting the number of 5 s bins each animal spent in an immobile posture. Results are presented as mean ± SEM and were analyzed by t-test (no significant differences detected). (TIF) [file pone.0070872.s001.tif]

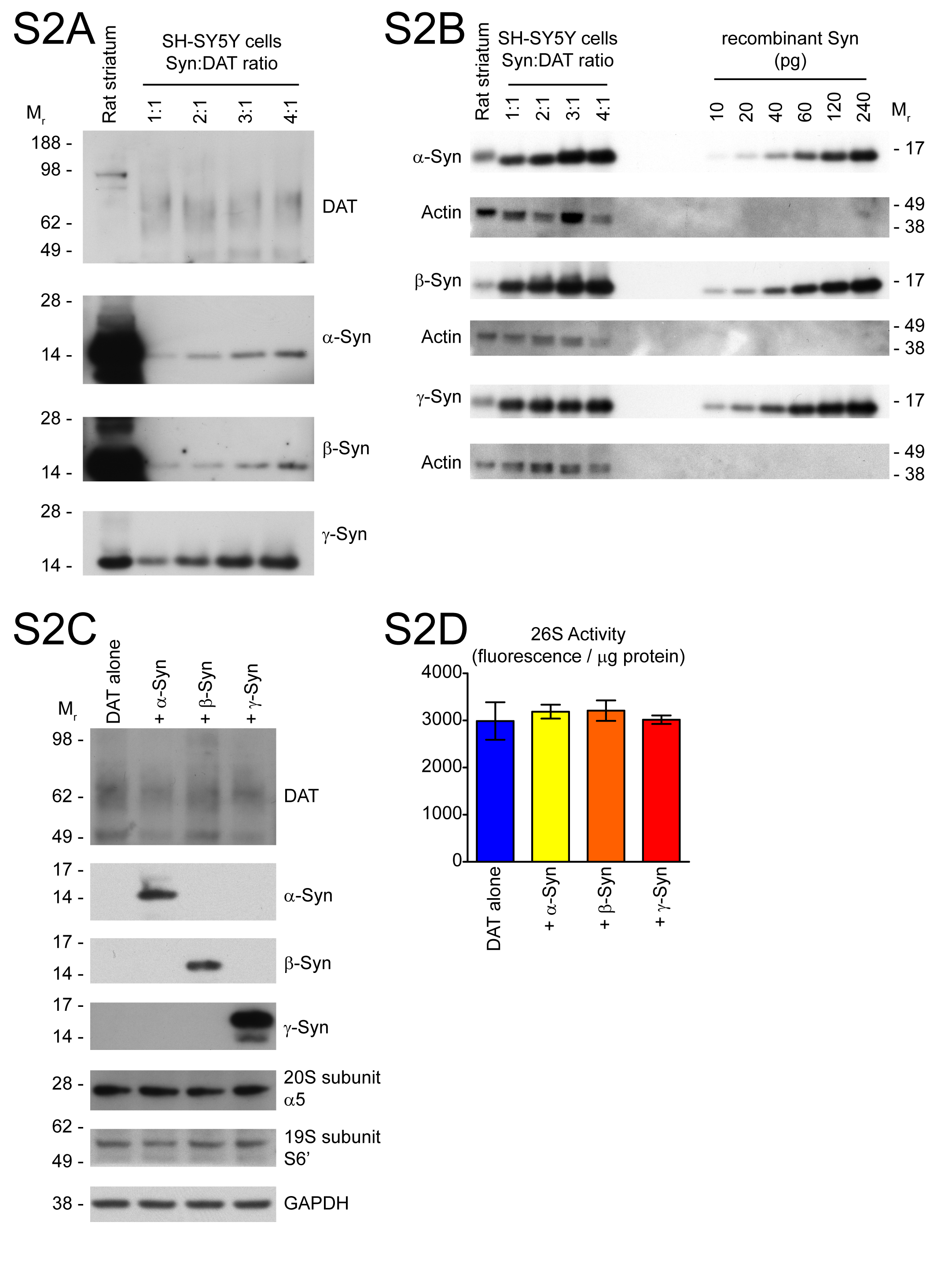

Supplement: Figure S2 — Cellular conditions associated with over-expression of synucleins. (A) Protein isolated from rat striatum and SH-SY5Y cells transfected with with 100 ng/cm2 DAT and 100–400 ng/cm2 of vector alone, α-Syn, β-Syn, or γ-Syn was analyzed by immunoblot. Protein loading was adjusted to generate equal immunodetection of DAT in each sample, and expression levels of α-Syn, β-Syn, and γ-Syn were compared between rat tissue and transfected cells. (B) Samples from A were adjusted for equal protein loading as indexed by actin immunoreactivity. Expression levels of α-Syn, β-Syn, and γ-Syn were compared between rat tissue and transfected cells. 10–240 pg/lane of purified recombinant α-Syn, β-Syn, or γ-Syn were immunoblotted simultaneously to estimate Syn abundance in each sample. (C) Expression levels of 19S proteasome subunit S6′ and 20S proteasome subunit α5 were assessed by immunoblot in cells transfected with 100 ng/cm2 DAT and 400 ng/cm2 of vector alone, α-Syn, β-Syn, or γ-Syn (n = 3). Syns and DAT were also probed to verify experimental conditions. GAPDH is displayed as a control for protein loading. Representative blot images from each condition are presented with approximate molecular mass of nearest protein ladder bands indicated (Mr). (C) Lactasystin-sensitive digestion of the model substrate suc-LLVYAMC was used to measure 26S proteasome activity in cells transfected as above. Proteasome activity is expressed as mean fluorescence ± SEM (excitation 355 nm; emission 460 nm). Data were analyzed by one-way ANOVA followed by Dunnet's post-hoc analysis for comparison to DAT-alone cells (n = 4; no significant differences detected). (TIF) [file pone.0070872.s002.tif]

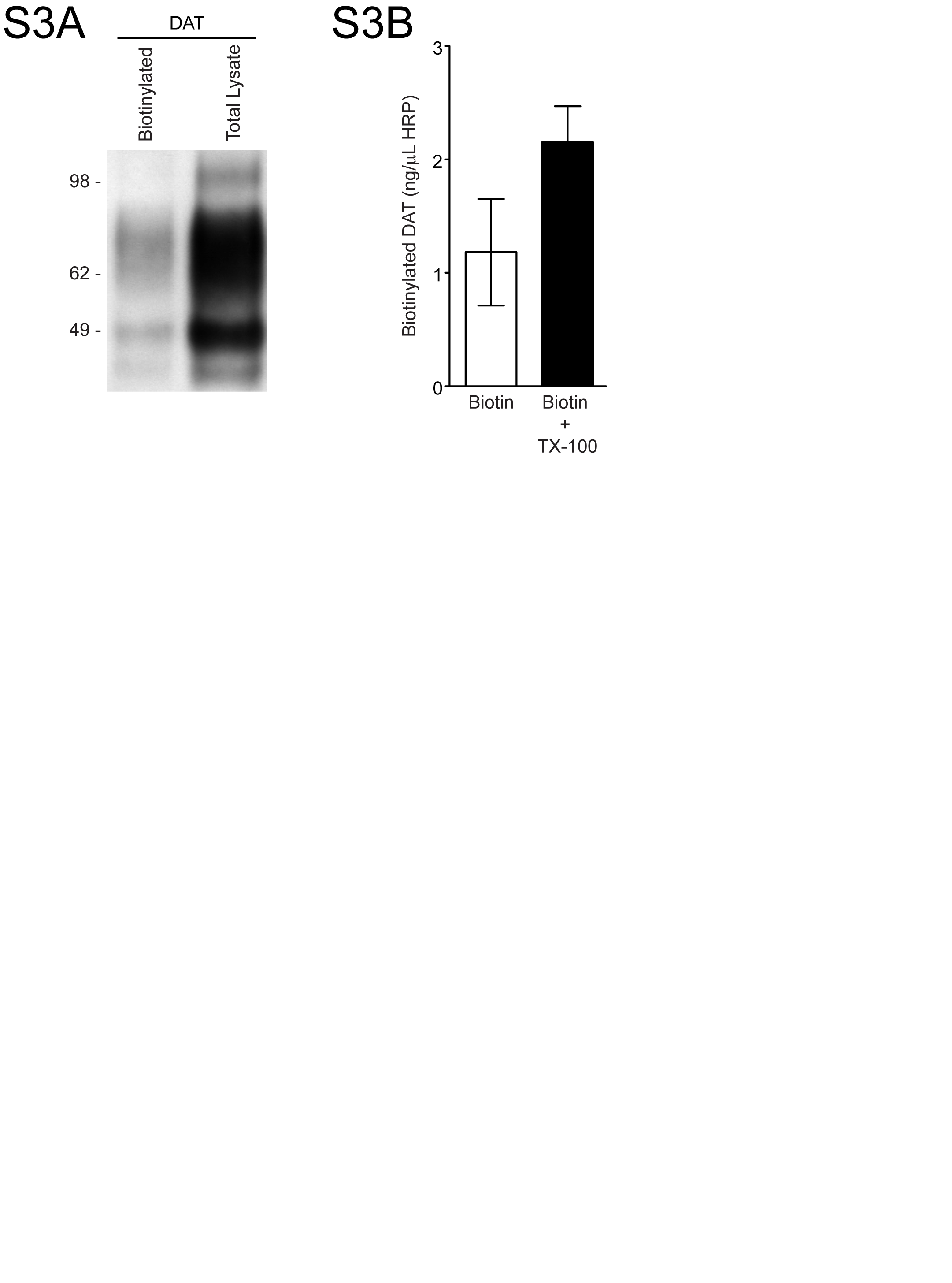

Supplement: Figure S3 — Distribution of DAT in transfected SH-SY5Y cells. (A) Direct comparison by immunoblot of DAT Total and DAT Biotin from cell surface biotinylation experiments (see also Fig. 1D). (B) Recovery of biotinylated DAT from intact or Triton X-100 permeabilized cells as quantified by o-phenyldiamine absorbance (see Supporting methods in File S1). (TIF) [file pone.0070872.s003.tif]

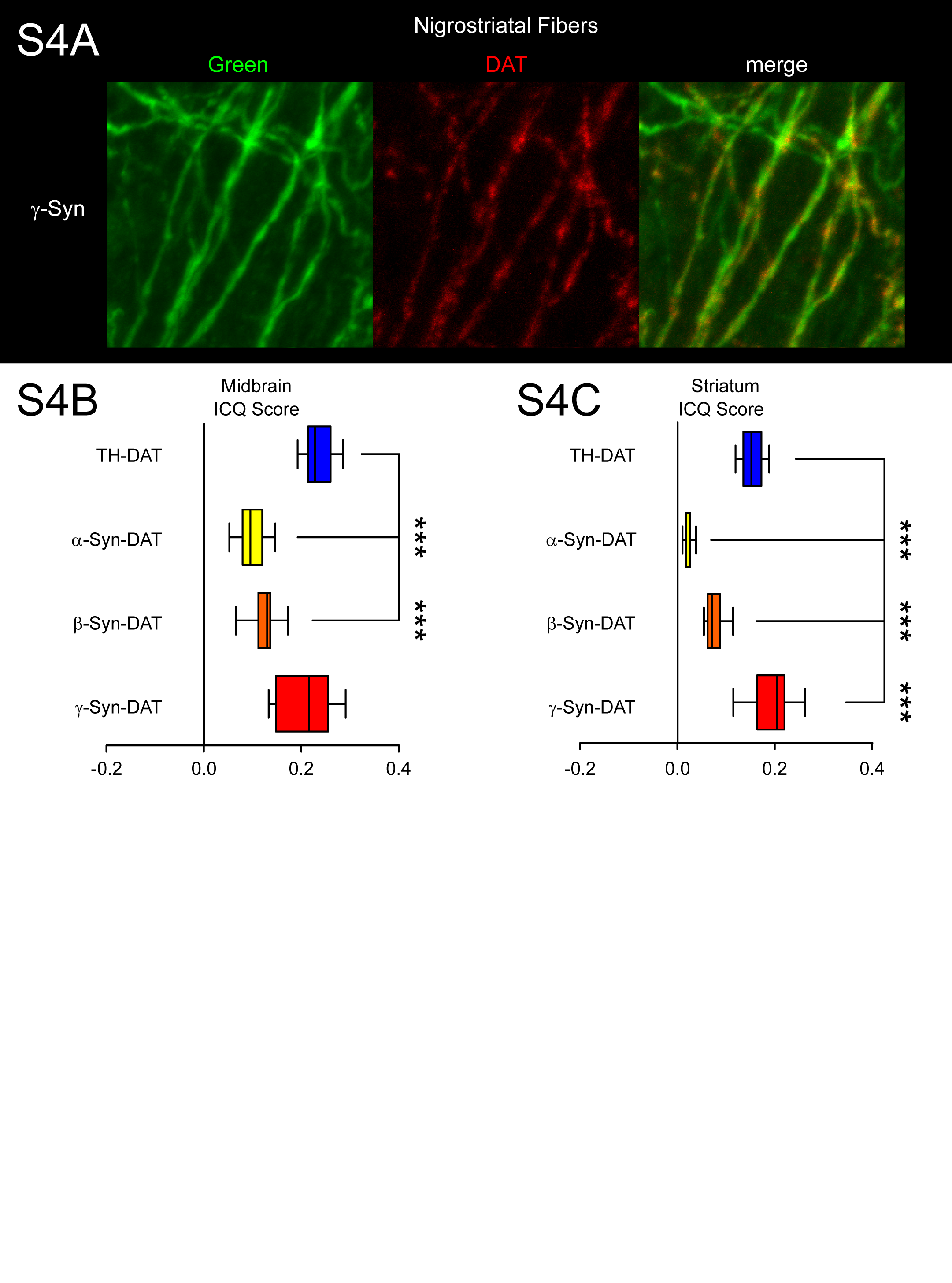

Supplement: Figure S4 — Synucleins and DAT in the brain. (A) Co-labeling of γ-Syn (green) and DAT (red) in dopaminergic fibers of the nigrostriatal pathway. Close correspondence of γ-Syn with DAT-positive structures is shown in the merge. Co-distribution of TH, α-Syn, β-Syn, and γ-Syn with DAT was assessed by intensity correlation analysis (ICA) in immunostained midbrain and striatal tissue (see Fig. 3A–3B for images). All Syn proteins and TH were positively co-distributed with DAT in these tissues (see Table S5 in File S1). Box-and-whisker plots (whiskers, 2.5th and 97.5th percentiles, boxes, 25th, 50th, and 75th percentiles) display ICQ values from (B) midbrain and (C) striatal fields analyzed for each pair. One-way ANOVA with Dunnett's post-hoc analysis was also used to compare Syn-DAT ICQ means to TH-DAT (***p<0.001). (TIF) [file pone.0070872.s004.tif]

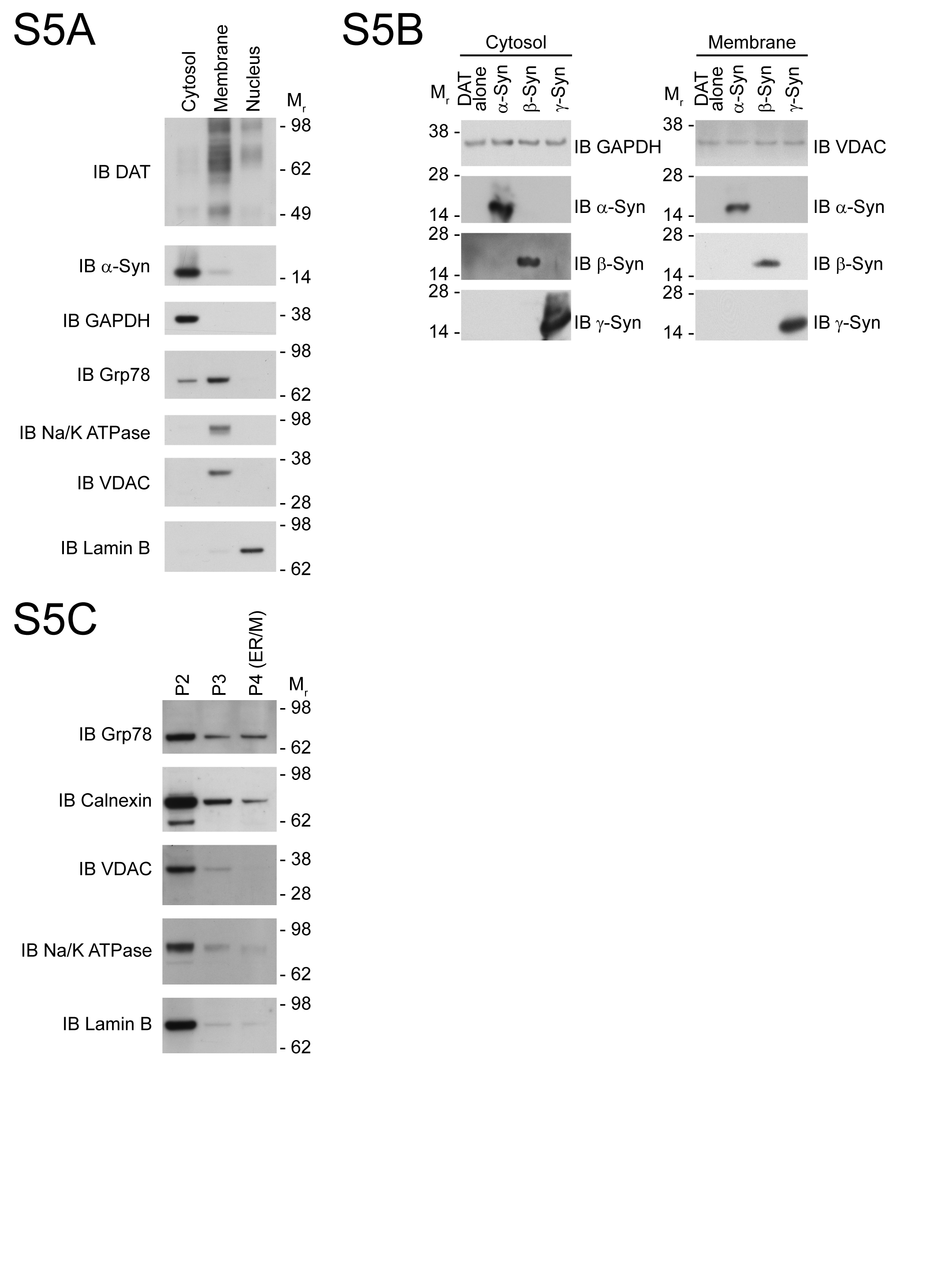

Supplement: Figure S5 — Cell fractionation and ER/M isolation controls. (A) SH-SY5Y cells were transfected with DAT (100 ng/cm2) and (400 ng/cm2) of α-Syn and cytosol, membrane, and nuclear fractions were prepared. Fractions were analyzed by immunoblot for expression of DAT, α-Syn, and markers for cytosol (glyceraldehyde 3-phosphate dehydrogenase, GAPDH), membrane (Grp78, Na/K ATPase, and voltage dependent ion channel, VDAC), and nuclear fractions (Lamin B). (B) Cytosol and membrane preparations from cells transfected with DAT (100 ng/cm2) and (400 ng/cm2) of empty vector, α-Syn, β-Syn, or γ-Syn were analyzed by immunoblot for expression of the Syns. (C) SH-SY5Y cells were harvested and processed for isolation of purified rough ER/M. Pellets (P2, P3, P4; see Materials and methods) were extracted in buffer containing detergents and analyzed by immunoblot for expression of markers for nuclear material (Lamin B), plasma membrane (Na/K ATPase), mitochondria (VDAC), and ER (Calnexin and Grp78). (TIF) [file pone.0070872.s005.tif]

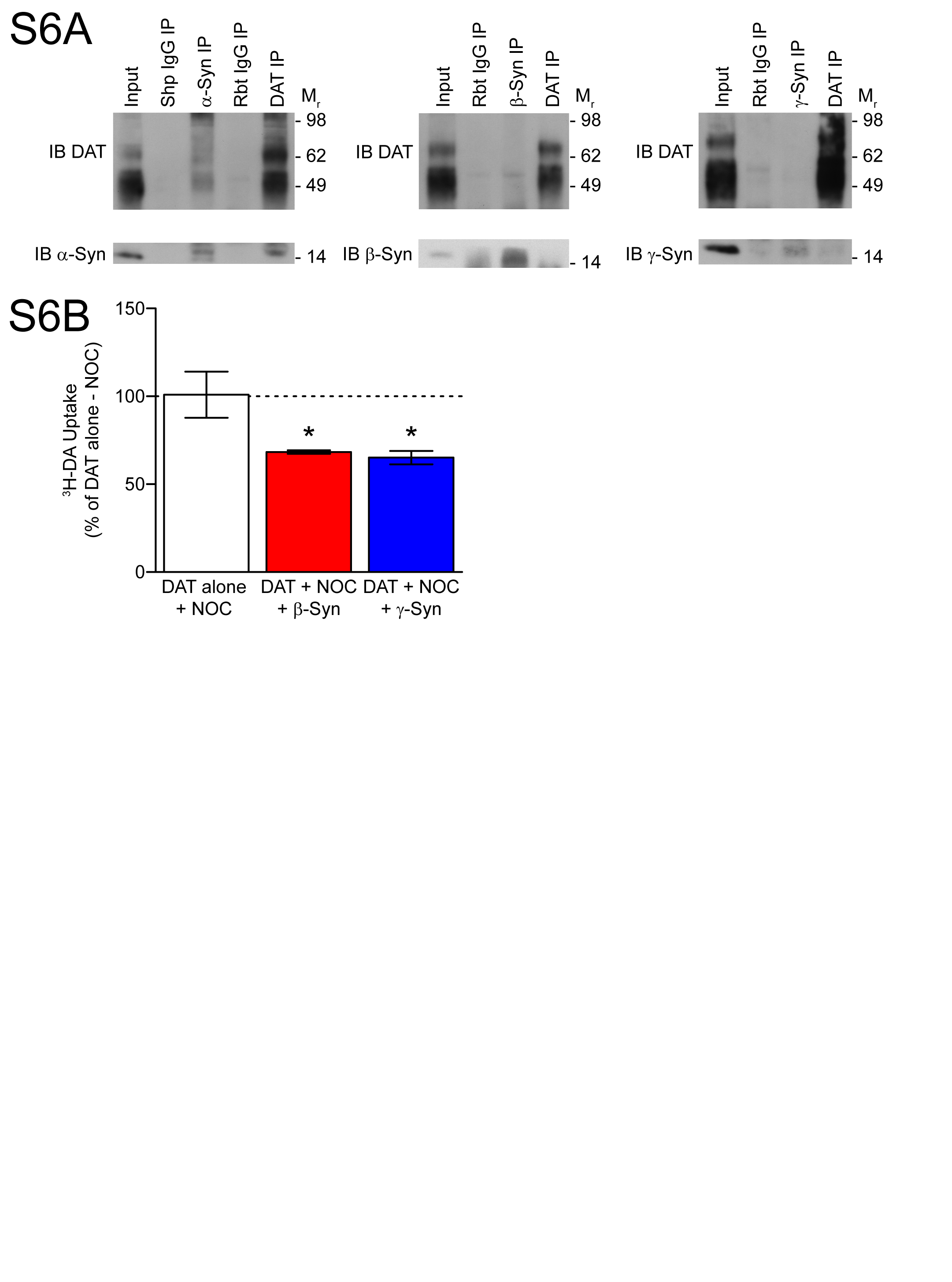

Supplement: Figure S6 — Analysis of interactions between synucleins and DAT. (A) SH-SY5Y cells were co-transfected with DAT and α-Syn, β-Syn, or γ-Syn and lysed for immunoprecipitation (IP) with antibodies (Table S1 in File S1) against Syns or DAT. IP from identical samples using pre-immune IgG (sheep, Shp IgG IP; rabbit, Rbt IgG IP) was performed in parallel as a control for specificity of each IP and co-IP. Input (5%) and IPs were analyzed by immunoblots probed (IB) for Syns and DAT. Blot images are representative of three independent experiments; faint non-specific bands were observed below 14 kDa on blots probed for β-Syn. (B) Uptake of [3H]-DA SH-SY5Y cells transfected with DAT (100 ng/cm2) and (400 ng/cm2) of empty vector, β-Syn, or γ-Syn was measured following treatment with 10 μM nocodazole (NOC) as described previously [12]. Values recorded from three assays performed in triplicate (mean ± SEM) are presented as percent of DAT alone (line at 100%). Non-specific uptake in the presence of 10 μM indatraline was subtracted. Uptake data were analyzed by t-test for difference from a theoretical mean of 100 (*p<0.05) and corrected for multiple t-tests [20]. (TIF) [file pone.0070872.s006.tif]
