# Supplementary material for: Synucleins Antagonize Endoplasmic Reticulum Function to Modulate Dopamine Transporter Trafficking
Source: PLoS One. 2013 Aug 13;8(8):e70872. doi: 10.1371/journal.pone.0070872 (PMC3742698; doi:10.1371/journal.pone.0070872)
Supplement: File S1 — Additional supporting information, including Supporting methods, results, tables, and references. (DOC) [file pone.0070872.s007.doc]

**Supporting information for:**

Synucleins antagonize endoplasmic reticulum function to modulate dopamine transporter trafficking

**Adam W. Oaks^1^, Nicholas Marsh-Armstrong^2,3^, Jessica M. Jones^1^, Joel J. Credle^1^, and Anita Sidhu^1^***

^1^Laboratory of Molecular Neurochemistry, Georgetown University Medical Center, Department of Biochemistry and Molecular & Cellular Biology, Washington, DC, USA

^2^The Solomon H. Snyder Department of Neuroscience, The Johns Hopkins University School of Medicine, Baltimore, MD, USA

^3^Hugo W. Moser Research Institute at Kennedy Krieger, Baltimore, MD, USA

*****To whom correspondence should be addressed: Anita Sidhu, PhD, Head, Laboratory of Molecular Neurochemistry, Georgetown University Medical Center, 3970 Reservoir Road, NW, The Research Building, Room W222, Washington, D.C. 20007, USA, Tel.: (202) 687-0282; Fax: (202) 687-0279; E-mail: sidhua@georgetown.edu

**Supporting methods**

**Animals**

All studies with animals were approved by the Georgetown University Institutional Animal Care and Use Committee (Protocol 10-076). α-Syn knockout mice (α-Syn KO) with homozygous deletion of exon 2 of *snca* [1] were crossed with F2 B6129X1 hybrids producing α-Syn heterozygous (α-Syn +/-) offspring (all founders purchased from Jackson Laboratories, Bar Harbor, ME), which were used to generate all experimental mice. Animals were genotyped by PCR on DNA purified from tail samples (Wizard SV, Promega, Madison, WI). Two reactions conducted with Bioline Taq DNA polymerase (Taunton, MA) as described previously [2] detected the intact snca gene (5’-CATTGGGTGGAATTTGAGC-3’ and 5’-GAGAGGAGTCAGTCTTTGG-3’) and the Neo insert of the deleted gene (5’-CTTGGGTGGAGAGGCTATTC-3’ and 5’-AGGTGAGATGACAGGAGATC-3’).

**Animal behavior**

Mice were subjected to an identical battery of behavior tests over a two week period, with at least 72 h between each test. Testing was conducted in a quiet (50–55 dB ambient noise), dedicated room. Locomotor activity (distance traveled) and anxiety-like behavior (center zone entries) were monitored for 10 min in the open field test (OFT, 40 x 40 cm with clear 35 cm high walls). Anxiety-like behavior on the elevated plus maze (EPM, 40 cm high, four 35 x 5 cm arms) was monitored for five min on mice placed at the intersection of open and closed arms. Behavior was analyzed as described previously [2] using the ANY-maze video tracking system (Stoelting, Wood Dale, IL) to count center zone (inner 25% of box) entries and distance traveled (OFT) and open arm entries (EPM). A previously described forced swim test (FST) protocol [3] was adapted for mice using a 15 cm glass cylinder filled to a depth of 13 cm with room temperature water. Mice were pre-tested for six min 24 h prior to the test period, then video recorded during the six min test period. Videos were scored by a blinded observer for the behavior present (swimming or immobility) in each five sec bin.

**Brain tissue protein extraction and fractionation**

For brain tissue collection animals were euthanized by decapitation and rapidly dissected on ice. To isolate the striatum a razor blade was first used to slice coronally through the brain at approximately Bregma -0.5, then alone the midline. Each half striatum was then dissected away from surrounding cortical tissues. Tissues were individually flash frozen in liquid nitrogen and stored at -80° C until protein extraction. Intact mouse striatal tissues were thawed and homogenized in ice cold buffer (10 mM Tris HCl, 100 mM NaCl, 1 mM EDTA, 1 mM EGTA, 250 mM sucrose, pH 7.4) with Complete Mini Protease Inhibitor Cocktail (Roche) and Halt Phosphatase Inhibitor Cocktail (Thermo Scientific, Rockford, IL). Homogenate protein concentration was determined by Lowry assay (Biorad), adjusted, and aliquots were frozen at -80° C until further extraction in complete buffer with the addition of detergents (1% Triton X-100, 0.5% Na-deoxycholate, 0.1% SDS) for 1 h at 4°C. To create membrane fractions un-extracted homogenate was thawed and centrifuged at 1,000 RCF for 5 min to pellet nuclei and debris. From this supernatant, a membrane fraction was isolated by centrifugation at 30,000 RCF for 45 min and protein was extracted in complete buffer as above.

**Immunoprecipitation**

Lysates for IP were prepared from co-transfected SH-SY5Y cells and IP was conducted as described previously [4] in a buffer (150 mM NaCl; 50 mM Tris HCl; 5 mM KCl; 1.5 mM CaCl_2_; 5 mM MgCl_2_) to which was added a ½ volume of 3% digitonin in H_2_O. Lysates were further extracted with the addition of 1.5 volumes of solubilization buffer (150 mM NaCl; 50 mM Tris HCl; 2 mM EDTA; 1% NP40; 1% Triton X-100; 0.5% sodium deoxycholate) for 30 min at 4 °C, and IP was conducted as described previously [4]. Briefly, lysates were pre-cleared with Protein A/G Plus-Agarose beads (Santa Cruz), then 2.5 μg of specific antibodies (Table S1) or non-immune control antibodies (sheep IgG, sc-2717; rabbit IgG, sc-2027; goat IgG, sc-2028; Santa Cruz) were added to 500 μL of cleared lysate (0.1 mg/mL protein) and incubated overnight at 4° C with rotation. 50 μL of beads were added to each IP for 1 h, pelleted at 7,700 RCF for 5 min, and washed 3 times with solubilization buffer. Protein was released with 70 μL of Laemmli sample buffer with 5% β-mercaptoethanol and analyzed by SDS-PAGE. Immunoprecipitation experiments were assessed qualitatively for enrichment relative to appropriate controls.

**Intensity correlation analysis**

Co-distribution of endogenous Syns and DAT was analyzed in the nigrostriatal system of WT mice. Each Syn-DAT pair was labeled via IHC (see Fig. 3 for images) and confocal images were captured of AlexaFluor 488 labeled Syn proteins and Alexafluor 568 labeled DAT. Tyrosine hydroxylase (TH; labeled with AlexaFluor 488) was used as a control for a protein known to co-distribute closely with DAT. Confocal images were captured on a Zeiss LSM 510 with constant imaging parameters applied to each channel (see Table S3 for additional details). Image stacks were sequentially captured with illumination at 488 nm and 543 nm (pixel size = 0.070 μm). Maximum projections were created from three horizontal optical sections spaced 1.2 μm apart to fully capture the fiber density and cell volume in the striatum and midbrain, respectively. A total of 24-29 images were analyzed from each section (8-13 from midbrain and 16 from striatum) and a total of 64 substantia nigra cells were analyzed. The intensity correlation analysis (ICA) module ([http://www.uhnresearch.ca/facilities/wcif/imagej/colour_analysis.htm#coloc_ica](http://www.uhnresearch.ca/facilities/wcif/imagej/colour_analysis.htm#coloc_ica)) was used in ImageJ to quantify the degree of co-distribution of each Syn-DAT pair (see Fig. 3 and Fig. S4 legend and Table S4). ICA output including PDM (product of the differences from the means) values, images, and plots were generated and the intensity correlation quotient (ICQ) was analyzed as described previously [5,6,7].

**Microplate quantification of biotinylated DAT**

A slightly modified procedure was used to isolate DAT from fixed cells labeled with sulfo-NHS biotin in the presence or absence of 0.3% Triton X-100 (see Fig. S3B). Biotinylated protein was isolated with streptavidin agarose beads as above, then beads were incubated with Rat-anti-DAT antibody (Table S1) overnight, washed, and incubated with Goat-anti-Rat HRP conjugated secondary (Santa Cruz). Bound HRP-conjugated secondary was quantified by colorimetric detection at 450 nm of the conversion of o-phenylenediamine in 96 well microplates.

**Supporting results**

**Neurochemical and behavioral status of α-Syn KO mice.**

To assess the contributions of β-Syn and γ-Syn to DAT trafficking *in vivo*, their expression, as well as the distribution and function of DAT, was examined in striata of α-Syn KO mice. In striatal lysates, β-Syn was significantly decreased (70 ± 7% of WT, p<0.05), with no other changes in γ-Syn or DAT expression (Fig. S1A). There were no changes in DAT expression in striatal membranes (Fig. S1A). Motor activity, anxiety-like, and depressive-like behaviors in α-Syn KO mice were assessed by the OFT, EPM, and FST to measure activity, anxiety-like, and depressive-like behaviors, respectively. No significant differences were detected in distance traveled or center zone entries on the OFT, open arm entries on the EPM, or immobility scores on the FST (Fig. S1B-S1C).

**Synuclein over-expression in SH-SY5Y cells achieves a normal Syn:DAT ratio**

In order to determine whether the over-expression of the Syns in transfected SH-SY5Y cells achieves a normal Syn:DAT ratio, the levels of the Syns at each Syn:DAT ratio were compared to protein isolated from rat striatum. When normalized to total DAT expression, immunoblot analysis showed that α-Syn and β-Syn are at a much higher ratio to DAT in the brain than in transfected cells, and that the γ-Syn:DAT ratio in the brain is similar to cells transfected with 4:1 γ-Syn:DAT (Fig. S2A). To achieve this ratio, the Syns are over-expressed at approximately 5-10 times normal levels in the brain (Fig. S2B). Syn-transfected SH-SY5Y cells have no loss of viability compared to DAT-alone cells (Fig. 1C). Proteasome activity, which can be impaired under conditions of extreme Syn over-expression [8,9], is unaltered in Syn-transfected SH-SY5Y cells (Fig. S2C-S2D).

**Intracellular localization of DAT**

DAT distribution was visualized in SH-SY5Y cells labeled with the ER-Tracker^TM^ dye (Molecular Probes, Invitrogen), a widely used means for monitoring the ER in living cells that provides specific labeling of ER membranes [10,11]. In imaged cells, both ER-Tracker^TM^ and DAT-mCherry signals moved together over time and throughout multiple imaging planes (see Videos S2-S3). DAT distribution was also compared in SH-SY5Y cells with the localization of VSVG (vesicular stomatis virus glycoprotein; VSVG-GFP). Live-cell imaging showed that VSVG was localized primarily in highly motile vesicles and at the cell periphery (see Video S4). The rapid movements observed with VSVG-GFP were distinct from DAT-mCherry (Video S4), which retained a more widely distributed and relatively static localization (see Videos S2-S3).

**Involvement of direct binding and microtubule tethering in synuclein modulation of DAT**

Our prior work has shown that α-Syn can modulate DAT trafficking through a direct protein-protein interaction [12,13,14]. In lysates from co-transfected cells, α-Syn antibodies co-immunoprecipitated (co-IP) DAT as expected. Surprisingly, however, IP of β-Syn or γ-Syn failed to bring down detectable levels of DAT (Fig. S6A). Modulation of DAT by α-Syn is reversible by treatment with the microtubule disrupting agent nocodazole [12], suggesting a model wherein α-Syn tethers DAT to the microtubule cytoskeleton to limit distribution of DAT to the cell surface. Modulation of NET trafficking by α-Syn is also microtubule-dependent [15,16], but trafficking of NET by β-Syn and γ-Syn is not [3] due to unknown differences in their mechanism of action. Similarly, exposure to nocodazole failed to reverse the negative modulation of DAT by β-Syn and γ-Syn (Fig. S6B). We were therefore unable to confirm the involvement of direct binding or microtubule tethering in the modulation of DAT by β-Syn and γ-Syn.

**Supporting tables**

**Table S1** Antibodies

| **Target** | **Source** | **Product Number** | **Host^a^** | **Dilutions** | | | |
| --- | --- | --- | --- | --- | --- | --- | --- |
|  |  |  |  | **IB** | **ICC** | **IHC** | **IP** |
| β-Actin | Santa Cruz | sc-1616 | Gt | 1:1000 | - | - | - |
| Calnexin | StressGen | ADI-SPA-865 | Rbt | 1:1000 | - | - | - |
| DAT | Millipore | MAB369 | Rt | 1:2000 | - | 1:200 | - |
| DAT | Millipore | AB5802 | Rbt | - | - | - | 1:60 |
| GAPDH | Cell Signaling | 2118 | Rbt | 1:3000 | - | - | - |
| Gpp130 | Covance | PRB-144C | Rbt | - | 1:300 | - | - |
| Grp78 | Santa Cruz | sc-1051 | Gt | 1:500 | - | - | - |
| Lamin B | Santa Cruz | sc-6216 | Gt | 1:1000 | - | - | - |
| Na/K ATPase | Abcam | ab7671 | Ms | 1:1000 | - | - | - |
| α-Syn | BD Transduction | 610787 | Ms | 1:2000 | - | 1:200 | - |
| α-Syn | Millipore | AB5334P | Shp | - | - | - | 1:200 |
| α-Syn | Santa Cruz | sc-7012 | Gt | - | 1:300 | - | - |
| β-Syn | Abcam | ab25650 | Rbt | 1:2000 | - | - | 1:200 |
| β-Syn | Novus Biologicals | NB100-79903 | Rbt | 1:1000^c^ | - | 1:200 | - |
| β-Syn | Santa Cruz | sc-9565 | Gt | - | 1:300 | - | - |
| γ-Syn | Abcam | ab55424 | Rbt | 1:2000 | - | - | - |
| γ-Syn | Abcam | ab47966 | Ms | 1:2000^d^ | - | - | - |
| γ-Syn | N. Marsh-Armstrong^e^ | N/A | Ms | - | - | 1:200 | - |
| γ-Syn | Santa Cruz | sc-10698 | Gt | - | - | - | 1:40 |
| γ-Syn | Santa Cruz | sc-10699 | Gt | - | 1:300 | - | - |
| TH | Santa Cruz | sc-25269 | Ms | - | - | 1:100 | - |
| VDAC | Cell Signaling | 4866 | Rbt | 1:1000 | - | - | - |

^a^Antisera raised in mouse (Ms), goat (Gt), rabbit (Rbt), rat (Rt), or sheep (Shp). ^b^Antibodies applied for immunoblots (IB), immunocytochemistry (ICC), immunohistochemistry (IHC), or immunoprecipitation (IP). ^c^Antibody used for detection of β-Syn from mouse brain by immunoblot. ^d^Antibody used for detection of γ-Syn by immunoblot following IP. ^e^Antibody provided as a generous gift by Nicholas Marsh-Armstrong [17].

**Table S2** Plasmids and constructs

| **Abbreviation** | **Insert^a^** | **Vector** | **Source^b^** | **Reference^c^** |
| --- | --- | --- | --- | --- |
| vector | - | pcDNA3.1 | Invitrogen | N/A |
| α-Syn | α-Syn | pcDNA3.1 | - | [18] |
| β-Syn | β-Syn | pcDNA3.1 | - | [3] |
| γ-Syn | γ-Syn | pcDNA3.1 | - | [3] |
| DAT | DAT | pcDNA3.1 | - | [18] |
| GFP vector | - | pCMV-AC-GFP | Origene | N/A |
| α-Syn-GFP | α-Syn | pCMV-AC-GFP | Origene | N/A |
| β-Syn-GFP | β-Syn | pCMV-AC-GFP | Origene | N/A |
| γ-Syn-GFP | γ-Syn | pCMV-AC-GFP | Origene | N/A |
| mCherry vector | - | pmCherry-N1 | Clontech | N/A |
| DAT-mCherry | DAT | pmCherry-N1 | - | N/A |
| tsVSVG-GFP | tsVSVG | pEGFP-N1 | Addgene plasmid 11912 | [19] |

^a^All inserts (except tsVSVG) consist of human cDNA sequences encoding the indicated proteins. GFP and mCherry constructs contain the same sequences with stop codons removed. All fluorescent protein tags indicated have been added to the carboxy terminal. ^b^Vectors and constructs not generated by sub-cloning (-) were originally purchased from Invitrogen (Carlsbad, CA), Origene Technologies (Rockville, MD), or Clontech Laboratories (Mountain View, CA), or acquired via the Addgene repository (Cambridge, MA). ^c^Un-cited constructs (N/A) were newly generated or purchased for this work. Additional details on cited constructs can be found in the referenced works.

**Table S3** Fluorochromes and optical configurations

| **Fluorochrome** | **Source** | **Absorbance**  **peak^b^** | **Laser^c^** | **Emission**  **peak^d^** | **Filters^e^** |
| --- | --- | --- | --- | --- | --- |
| TurboGFP^a^ | OriGene | 482 nm | 488 nm | 502 nm | BP 500-530 |
| EGFP | Clontech | 490 nm | 488 nm | 508 nm | BP 500-530 |
| mCherry | Clontech | 587 nm | 543 nm | 610 nm | BP 565-615 |
| Alexa Fluor 488 | Invitrogen | 499 nm | 488 nm | 520 nm | BP 500-550 |
| Alexa Fluor 568 | Invitrogen | 578 nm | 543 nm | 603 nm | BP 565-615 |
| Alexa Fluor 633 | Invitrogen | 631 nm | 633 nm | 650 nm | BP 650-710 |
| DAPI | Southern Biotech | 358 nm | 710 nm (2P) | 455 nm | BP 435-485 |
| ER Tracker Blue-White | Invitrogen | 372 nm | 800 nm (2P) | 555 nm | KP 685 |

^a^All GFP-tagged proteins were labeled with TurboGFP except VSVG-GFP. ^b^Fluorescent properties as reported by manufacturer or vendor. ^c^Laser lines are as described in Materials and methods. Two-photon illumination (2P) with Coherent Chameleon XR Ti:Sapphire laser was at indicated wavelengths. ^d^Fluorescent properties as reported by manufacturer or vendor. ^e^Filter sets used on LSM 510 as described by Zeiss software.

**Table S4** ICA-ICQ^a^ analysis of Syn-DAT co-localization in the brain

|  | **Stain**^b^ | **Median ICQ^c^** | **Sign test^d^** | **Mean ICQ ± SEM^e^** | **t-test^f^** | **n** |
| --- | --- | --- | --- | --- | --- | --- |
| **Midbrain** | TH-DAT | 0.2280 | >0 ** | 0.2343 ± 0.0097 | ≠ 0 *** | 9 |
|  | α-Syn-DAT | 0.0950 | >0 ** | 0.0994 ± 0.0082 | ≠ 0 *** | 12 |
|  | β-Syn-DAT | 0.1300 | >0 *** | 0.1244 ± 0.0073 | ≠ 0 *** | 13 |
|  | γ-Syn-DAT | 0.2150 | >0 ** | 0.2081 ± 0.0205 | ≠ 0 *** | 8 |
| **Striatum** | TH-DAT | 0.1520 | >0 *** | 0.1538 ± 0.0055 | ≠ 0 *** | 16 |
|  | α-Syn-DAT | 0.0180 | >0 *** | 0.0207 ± 0.0019 | ≠ 0 *** | 16 |
|  | β-Syn-DAT | 0.0705 | >0 *** | 0.0750 ± 0.0040 | ≠ 0 *** | 16 |
|  | γ-Syn-DAT | 0.2035 | >0 *** | 0.1926 ± 0.0100 | ≠ 0 *** | 16 |

^a^Intensity correlation analysis (ICA) results generated from PDM values as described in Supplemental methods. ^b^Sagittal sections from WT mice were co-stained with each Syn-DAT pair, or with TH and DAT, as described in Materials and methods. ICA-ICQ analysis quantified co-distribution of the listed pairs. ^c^Global median intensity correlation quotients (ICQ) of all images analyzed. ^d^Wilcoxon signed rank test against hypothetical median of zero (**=p<0.01; ***=p<0.001). ^e^Global mean ICQ of all images or cells analyzed. ^f^Two tailed t-test against hypothetical mean of zero (***=p<0.001).

**Supporting references**

1. Abeliovich A, Schmitz Y, Farinas I, Choi-Lundberg D, Ho WH, et al. (2000) Mice lacking alpha-synuclein display functional deficits in the nigrostriatal dopamine system. Neuron 25: 239-252.

2. Graham DR, Sidhu A (2010) Mice expressing the A53T mutant form of human alpha-synuclein exhibit hyperactivity and reduced anxiety-like behavior. J Neurosci Res 88: 1777-1783.

3. Jeannotte AM, McCarthy JG, Redei EE, Sidhu A (2009) Desipramine modulation of alpha-, gamma-synuclein, and the norepinephrine transporter in an animal model of depression. Neuropsychopharmacology 34: 987-998.

4. Lee FJ, Liu F, Pristupa ZB, Niznik HB (2001) Direct binding and functional coupling of alpha-synuclein to the dopamine transporters accelerate dopamine-induced apoptosis. FASEB J 15: 916-926.

5. Li Q, Lau A, Morris TJ, Guo L, Fordyce CB, et al. (2004) A syntaxin 1, Galpha(o), and N-type calcium channel complex at a presynaptic nerve terminal: analysis by quantitative immunocolocalization. J Neurosci 24: 4070-4081.

6. Matthies H, Han Q, Shields A, Wright J, Moore J, et al. (2009) Subcellular localization of the antidepressant-sensitive norepinephrine transporter. BMC Neuroscience 10: 65.

7. Zhang ZW, Burke MW, Calakos N, Beaulieu JM, Vaucher E (2010) Confocal analysis of cholinergic and dopaminergic inputs onto pyramidal cells in the prefrontal cortex of rodents. Front Neuroanat 4: 21.

8. Snyder H, Mensah K, Theisler C, Lee J, Matouschek A, et al. (2003) Aggregated and monomeric alpha-synuclein bind to the S6' proteasomal protein and inhibit proteasomal function. J Biol Chem 278: 11753-11759.

9. Snyder H, Mensah K, Hsu C, Hashimoto M, Surgucheva IG, et al. (2005) beta-Synuclein reduces proteasomal inhibition by alpha-synuclein but not gamma-synuclein. J Biol Chem 280: 7562-7569.

10. Denoyelle C, Abou-Rjaily G, Bezrookove V, Verhaegen M, Johnson TM, et al. (2006) Anti-oncogenic role of the endoplasmic reticulum differentially activated by mutations in the MAPK pathway. Nat Cell Biol 8: 1053-1063.

11. Irshad S, Mahul-Mellier AL, Kassouf N, Lemarie A, Grimm S (2009) Isolation of ORCTL3 in a novel genetic screen for tumor-specific apoptosis inducers. Cell Death Differ 16: 890-898.

12. Wersinger C, Sidhu A (2005) Disruption of the interaction of alpha-synuclein with microtubules enhances cell surface recruitment of the dopamine transporter. Biochemistry 44: 13612-13624.

13. Wersinger C, Prou D, Vernier P, Niznik HB, Sidhu A (2003) Mutations in the lipid-binding domain of alpha-synuclein confer overlapping, yet distinct, functional properties in the regulation of dopamine transporter activity. Mol Cell Neurosci 24: 91-105.

14. Wersinger C, Prou D, Vernier P, Sidhu A (2003) Modulation of dopamine transporter function by alpha-synuclein is altered by impairment of cell adhesion and by induction of oxidative stress. FASEB J 17: 2151-2153.

15. Jeannotte AM, Sidhu A (2007) Regulation of the norepinephrine transporter by alpha-synuclein-mediated interactions with microtubules. Eur J Neurosci 26: 1509-1520.

16. Jeannotte AM, Sidhu A (2008) Regulated interactions of the norepineprhine transporter by the actin and microtubule cytoskeletons. J Neurochem 105: 1668-1682.

17. Nguyen JV, Soto I, Kim KY, Bushong EA, Oglesby E, et al. (2011) Myelination transition zone astrocytes are constitutively phagocytic and have synuclein dependent reactivity in glaucoma. Proc Natl Acad Sci U S A 108: 1176-1181.

18. Wersinger C, Sidhu A (2003) Attenuation of dopamine transporter activity by alpha-synuclein. Neurosci Lett 340: 189-192.

19. Presley JF, Cole NB, Schroer TA, Hirschberg K, Zaal KJ, et al. (1997) ER-to-Golgi transport visualized in living cells. Nature 389: 81-85.

20. Bland JM, Altman DG (1995) Multiple significance tests: the Bonferroni method. BMJ 310: 170.
